# Shear performance of slender reinforced concrete beams: an analysis of various international standards

**DOI:** 10.1038/s41598-026-50769-8

**Published:** 2026-05-15

**Authors:** Sabry Fayed, Ali Basha, Amira Elnagar

**Affiliations:** 1 Civil Engineering Department, Faculty of Engineering, Kafrelsheikh University, Kafrelsheikh, Egypt; 2 Civil Engineering Department, Higher Institute of Engineering, King Mariot, Alexandria, Egypt

**Keywords:** Reinforced concrete, Slender beams, Shear reinforcement, Shear capacity, Engineering, Materials science, Mathematics and computing

## Abstract

The performance and design of reinforced concrete, also known as slender beams are explained in the current study by a number of international codes. Slender beams are essential in modern construction because they preserve structural integrity while allowing for efficient material use. This research conducts a comprehensive comparative analysis of shear design provisions for slender reinforced concrete beams across major international standards, including ECP, ACI, Eurocode, CSA, BS, and JSCE. The innovation lies in systematically identifying differences and commonalities in methodologies related to shear capacity determination, minimum reinforcement requirements, and serviceability criteria. By addressing the current gap of exhaustive analyses needed to standardize criteria and resolve methodological discrepancies, the work aims to enhance the reliability, effectiveness, and uniformity of global shear design processes for slender beams. This comparative approach is intended to assist engineers and academics in making more informed design decisions, thereby improving the efficiency, structural integrity, and safety of slender beam applications in modern construction. In summary, the novel contribution is the exhaustive and systematic comparison of various international design codes concerning slender RC beam shear design. Identification of inconsistencies and variations that affect structural security and design efficiency. Providing insights that could aid in harmonization and standardization of shear design provisions worldwide. Offering recommendations towards achieving more consistent, safe, and economical slender beam designs in contemporary construction.

## Introduction

Research into the shear strength of RC slender beams remains vital, internationally as well as nationally, due to difficulty of the shear problem. In structural concrete, shear is a complicated problem. Critical shear cracks, which typically develop abruptly, serve as an indication of shear failure in slender reinforced concrete (RC) columns lacking shear reinforcement. Since then, more approaches for designing shear-resistant members have been established as investigated by^1–7^. A beam is classified as RC slender if its span-to-height ratio is ≥ 5 or its shear-span ratio is ≥ 2.5. An RC slender beam is characterized by a relatively high shear span-to-depth ratio (a/d) or span-to-height ratio (a/h), although various articles and codes offer varying definitions for this category of beam. For example, in Kani^8^ and ACI 318^11^, a/d > 2.5; in JSCE^9^, a/d > 2; and in EC2^12^, a/h > 3 as shown in Table 1. When a component defects, it is typically brittle and has low ductility. The component’s stress profile becomes quite complex following oblique cracks. The presumption of a plane region for shear stress in a typical homogeneous elastic body is useless due to significant stress distribution during the failure process of the constituent^10^. It is essential for enhancing forecast precision based on its mechanical mechanism. Slender beams with reinforced concrete are vital structural elements used in the construction of concrete structures due to their facilitate load distribution and transfer within the framework. Their significance is especially clear in engineering and architectural applications, where they enable flexible and visually beautiful designs by providing the support essential for large spans. British Standards (BS), Eurocode, and the American Concrete Institute (ACI) represent the international codes from which standards for the design of slender beams are derived. The objectives of these codes are to enhance the beams’ resistance to failure, control deflections, and guarantee structural safety.

To guarantee structural safety and performance, many design rules impose specific limitations on slender reinforced concrete beams. Numerous critical elements regarding the boundaries are delineated in significant codes^11–16^ as shown in Table 2. The constraints include aspect ratio, depth-to-span ratio, minimum reinforcement specifications, and deflection limitation. The aspect ratio, characterized by the proportion of the beam’s length to its effective depth, is essential in influencing its performance under loading. The depth-to-span ratio quantifies the beam’s depth relative to its span, which is crucial for structural stability. Minimum reinforcement standards are set to guarantee sufficient strength and ductility, hence averting failure under diverse loading scenarios. Moreover, regulating deflection is essential; constraining deflection under service loads is crucial for preserving the functioning and utility of the structure. These characteristics jointly enhance the overall efficiency as well as reliability of beam constructions. These regulations provide essential guidelines for the analysis and design of slender beams, ensuring their efficacy and safety across various applications.

**Table 1:** Definitions of slender RC beams according to different international standards.

Code ID	Definition
(ACI) 318^11^	Shear span-to-depth ratio (a/d) > 2.5
(EN 1992)^12^	Span-to-height ratio (a/h) > 3
(BS 8110)^13^	Shear span-to-depth ratio (a/d) > (2.0–2.5)
(CSA A23.3)^14^	Shear span-to-depth ratio (a/d) > 2.5
(JSCE)^9^	Shear span-to-depth ratio (a/d) > 2
ECP^17^	Span-to-height ratio (L/h) ≥ 5 Or Shear span-to-depth ratio (a/d) ≥ 2.5

**Table 2:** A comprehensive summary of slender beam code limits.

Code ID	Maximum aspect ratio	Depth to span ratio	Minimum reinforcement	Deflection control	Crack control
(ACI) 318^11^	20	Simply supported beams: L/12 for deflection control	Tension reinforcement: minimum area of 0.002 of the gross concrete area	Total loads are L/240, while live loads are L/360.	Limits on crack width are provided based on exposure conditions
(EN 1992)^12^	12	Recommended limit of L/15 for deflection	should not be less than 0.1% of the cross-section	Limits of L/250 for serviceability	Limits on crack width for durability considerations
(BS 8110)^13^	12	Limit of L/12 for simply supported beams	Minimum area of tension reinforcement is 0.2% of the gross area.	Maximum deflection limits: L/360 for live loads	Limiting crack widths based on environmental exposure
(CSA A23.3)^14^	No strict limit	Recommended limit of L/12 for deflection	A minimum of 0.15% of the total area must be reinforced by tension.	L/360 for live loads	regulating crack widths based on exposure variables.
(IS 456:2000)^15^	12	L/20 for simply supported beams	Minimum tension reinforcement should be 0.12% of the cross-section.	Limits of L/300 for deflections	Allowable crack widths for durability
(AS 3600)^16^	No explicit limit	Recommended limit of L/12 for deflection	0.2% of the total area is the minimum area needed for longitudinal reinforcement.	Maximum deflection limits of L/250 for service loads	Limiting crack widths in aggressive environments

The prevailing understanding of slenderness in ECP is that flexural behavior dominates over shear and second-order effects. A beam is considered slender when it possesses a span-to-depth ratio (L/d) that defines its slenderness. In such instances, the beam’s behavior is predominantly influenced by flexural action instead of shear or compressive forces. Additionally, for slender beams, concerns regarding instability or buckling are not significant, particularly in the context of deep beams or columns^17,18^. This classification highlights the distinct performance characteristics of slender beams in structural applications. The term “slender beam” is implied in the ECP through deflection control and design assumptions rather than being explicitly defined as in the EC2 or ACI.

An RC beam’s resilient to shear comprises of both beam and arch motion. When tension is transferred into steel, beam action happens based on the link between steel and concrete. Bonding slip and cracking along the beam length impede beam action during loading, permitting the load to continuously generate shear by arch action. Arch action predominates in short or wide beams, while beam action is more prevalent in slender beams. Regrettably, shear resistance in a reinforced concrete beam arises from a mixture of bond slippage and cracking, which inhibit the full bond force necessary to maintain beam action from forming^19^. In order to further investigate the shear behavior of RC beams lacking diagonal reinforcement, three shear transfer mechanisms can be considered: dowel action of longitudinal reinforcement, shear transfer through aggregate interlock, and shear tensions within the flexural compression zone or uncracked concrete^20^. In all, 48 RC beams devoid of transverse reinforcement were assessed by^21^ both at room temperature and at extreme heat. The evaluated parameters encompassed the ambient temperature, the shear-span-to-depth ratio (a/d), and the tensile reinforcement ratio (ρ). Research revealed that shear strength loss following exposure to high temperatures was evident in beams with a smaller amount of tension reinforcement. Conversely, the shear strength reduction of the beams exhibited minimal sensitivity to variations in the shear span-to-depth ratio (a/d).

**Fig. 1:**
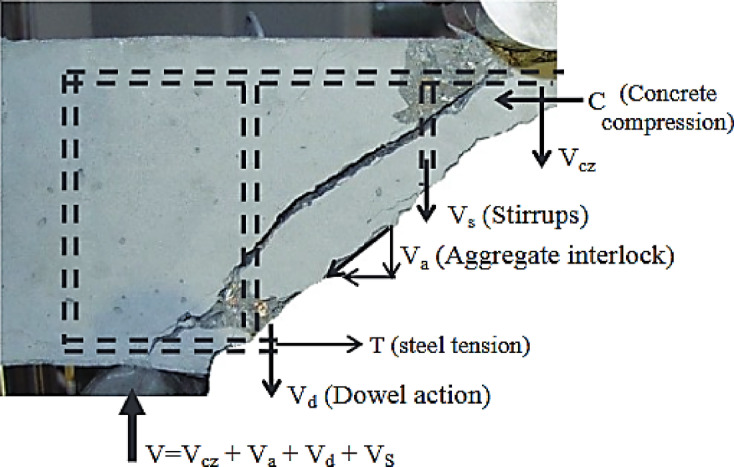
Transverse reinforcement for shear stresses in a beam: a mechanism^28^.

In the majority of contemporary shear analysis design approaches, a reinforced concrete beam’s shear strength can be estimated by adding the concrete contribution ($\:{v}_{c}$ = $\:{v}_{cz}$ + $\:{v}_{a}$ + $\:{v}_{d}$) as well as the role of transverse reinforcement ($\:{v}_{s}$), as seen in Fig. 1. A beam without transverse reinforcement endures shear, which is referred to the concrete contribution^26,28–37^. In slender beams, shear is predominantly countered through beam action, wherein internal forces are conveyed through a combination of flexural and shear stresses spread along the member. In this instance, factors such as aggregate interlock across inclined cracks, dowel action of the longitudinal reinforcement, and the contribution of the uncracked compression zone are crucial in reducing shear. Aggregate interlock offers resistance via the rough surfaces of cracks, whereas dowel action facilitates shear transfer through bending and shear in the reinforcing bars. Furthermore, the compression zone facilitates the transmission of diagonal compressive forces. Conversely, in non-slender beams (or deep beams), arch action prevails as the primary mechanism, whereby stresses are conveyed directly to the supports via inclined compression struts, hence diminishing dependence on shear reinforcement and crack-related mechanisms. The shift from beam action to arch action elucidates the discrepancies in shear capacity forecasts among design programs and underscores the necessity of precisely representing these dynamics in analytical models and design formulations. Equation 1 shows the transverse reinforcement yields, which convert to a shear force.

1 $$\:{v}_{s}=\frac{{A}_{v}*{f}_{vy}*d\:}{s}$$

Equation 2 shows the yields from transverse reinforcement that correspond to shear stress.

2 $$\:\tau\:=\frac{{A}_{v}*{f}_{vy}\:}{b*s}\:={\:\rho\:}_{v}\mathrm{*}\:{f}_{vy}$$

Where $\:{A}_{v}$, s and $\:{f}_{vy}$ denote for the transverse reinforcement’s area, spacing, and yield strength, respectively.

The main aim of this research is to perform a comprehensive analysis and comparison of the shear design provisions for slender reinforced concrete beams as stipulated by various international codes, including ECP, ACI, Eurocode, CSA, BS, and JSCE. The purpose of this research is to identify differences and commonalities to facilitate standardization, enhance the reliability, effectiveness, and uniformity of shear design processes worldwide. Although multiple international standards offer guidelines for the shear design of slender reinforced concrete beams, there is an absence of exhaustive comparative analyses emphasizing the distinctions and commonalities among these codes. Current research often focuses on specific codes or simplified models, resulting in a gap in understanding the broader implications of these variations on structural security and design efficacy. There is a lack of proven, standardized criteria that can be widely applied for the shear design of RC slender beams reinforced concrete beams, particularly in light of the swift advancement of new materials and building technology. Moreover, few studies have investigated the influence of these inconsistencies on actual structural reliability and cost-effectiveness under various environmental and loading situations.

The design of slender reinforced concrete beams is critical in construction engineering, necessitating precise and reliable methodologies to ensure durability and service life. Significant inconsistencies and methodological differences among global shear standards for design create challenges for engineers, potentially resulting in excessively conservative designs that increase costs or insufficient reinforcement that jeopardizes safety. This variability affects the selection of suitable design standards and may result in inconsistencies in structural performance across various geographical regions. Therefore, systematically evaluating and addressing these disparities is essential to establish standardized, efficient, and safe shear design approaches for slender reinforced concrete beams worldwide.

## Slender beams and non slender beams

### Slender beams

**Fig. 2:**
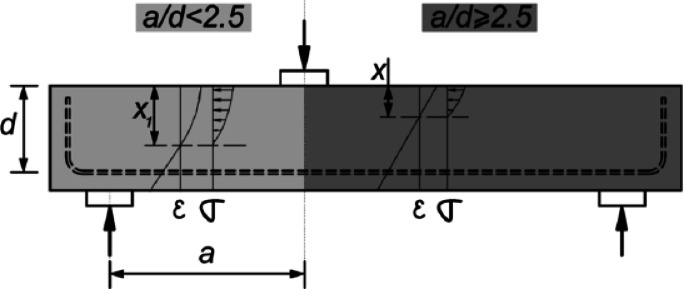
The distributions of stress and strain in both slender and non-slender shear spans, as well as the neutral axis depth^29^.

Figure 2 shows that the slender reinforced concrete beams are typically characterized by a shear span-to-depth ratio greater than 2.5. These beams are designed to maximize material efficiency without compromising structural integrity. Because of their dimensions, slender RC beams are more prone to lateral-torsional buckling and need to be carefully designed to guarantee sufficient flexural and shear strength.

### Non slender beams

The shear span-to-depth ratio of non-slender concrete- reinforced beams is typically less than 2.5, as shown in Fig. 2. These beams are more durable and buckling-resistant because they usually have bigger cross-sectional dimensions. Non-slender beams are typically constructed with distinct considerations for load-bearing capacity and deflection, and they typically rely more on the concrete itself for shear resistance.

Table 3 outlines the attributes of slender reinforced concrete (RC) beams in contrast to non-slender RC beams, emphasizing critical aspects such as definition, structural behavior, relevance, and design ramifications. Slender reinforced concrete beams are characterized by a shear span-to-depth ratio above 2.5, resulting in heightened vulnerability to buckling and augmented deflections, while exhibiting enhanced resistance to shear failure. In contrast, non-slender reinforced concrete beams possess a shear span-to-depth ratio of less than 2.5, providing enhanced shear resistance and stability. The importance of RC slender beams is attributed to their increased susceptibility to shear failure, requiring meticulous attention to shear reinforcement in their design. This meticulous technique differs from the more straightforward design strategies suitable for non-slender beams, which are typically reliable and grounded in proven design equations. Moreover, RC slender beams may encounter challenges with lateral stability and necessitate more intricate analytical approaches, whereas non-slender beams often exhibit reliable performance when well built. Slender beams are frequently utilized in constructions like bridges and skyscrapers, whilst non-slender beams are typically applied in building foundations and parking facilities.

**Table 3:** Comparison between slender and non slender RC beams.

Feature	Slender RC beams	Non slender RC beams
Definition	Shear span to depth ratio (a/d ≥ 2.5)	Shear span to depth ratio (a/d ˂2.5)
Structural behavior	1- Greater buckling susceptibility.	1- More resistant to buckling.
	2- Increased deflections while under stress.	2- Decreased deflections.
Significance	Greater shear stress and the possibility of shear failure can result from higher ratios.	More resistance to shear and stability are offered by lower ratios.
Design implications	Requires careful consideration of shear reinforcement to prevent failure.	Simple design that relies on the cross-section of the beam to withstand shear.
Performance	1- Affected by lateral stability.	1- Performance that is predictable using well-established design formulas.
	2- Demands sophisticated analysis methods.	2- Behavior that is simple under typical loading circumstances.
	3- May experience significant shear-related issues if improperly designed.	3- Usually performs dependably when using predetermined design standards.
Applications	Used in bridges, high-rise buildings	Common in foundations, parking structures

## Shear design for slender beams

### RC slender beams in ECP 203–2020

For RC slender beam shear design, the Egyptian Code provides a comprehensive foundation. In order to guarantee that beams are capable of withstanding shear forces while keeping structural integrity and serviceability, It emphasizes how concrete and shear reinforcement contribute to shear strength. The overall shear strength of a beam () can be determined using Eq. 3.

3 $$\:{v}_{u}={v}_{c}+\:{v}_{s}$$

Where $\:{v}_{s}$ is the shear reinforcement provides the shear strength in (kN) and $\:{v}_{c}$ is the concrete’s shear strength in (kN). The effective depth of the beam (d) in (mm) and the concrete’s typical compressive strength ($\:{f}_{cu}$) in (MPa) are used for determining the shear strength of the concrete. The Egyptian code provides tables and formulas that help in calculating $\:{v}_{c}$ according to the beam’s dimensions and material properties.

#### Concrete contribution ($\:{\boldsymbol{v}}_{\boldsymbol{c}}$)

Concrete contribution can be calculated from Eq. 4.

4 $$\:{v}_{c}=0.16*\:\sqrt{{f}_{\boldsymbol{c}\boldsymbol{u}\:}}*b*d$$

Where b is the beam’s web width in (mm), d is the effective depth of the beam in (mm), and f_cu_ is the concrete characteristic cube strength in (MPa). This formula is applicable to slender beams that have no axial compression or tension and sufficient longitudinal tension reinforcement. Dowel action and aggregate interlock should be able to withstand normal amounts of shear cracking. You can figure out how much the shear support contributes by using Eq. 5.

#### Shear reinforcement contribution ($\:{\boldsymbol{v}}_{\boldsymbol{s}})$

Equation 5 can be used to estimate the shear reinforcement contribution.

5 $$\:{v}_{s}=\frac{{A}_{sv}*{f}_{y}*d\:}{s}*\mathrm{c}\mathrm{o}\mathrm{t}\left({\uptheta\:}\right)$$

Where $\:{A}_{sv}$ is area of one stirrup leg х number of legs in (mm²), d implies the beam’s effective depth in (mm), s implies the spacing in mm between stirrups in (mm), $\:{f}_{y}$ represents the stirrups’ yield strength in (MPa), typically 240 or 360 MPa, θ is the angle of the concrete compression strut, usually assumed to be 45°, and cot θ = 1.

The ECP code specifies that the concrete contribution to shear is limited to $\:{q}_{cu}=0.24*\:\sqrt{{f}_{\boldsymbol{c}\boldsymbol{u}\:}}$​​, Where $\:{f}_{\boldsymbol{c}\boldsymbol{u}\:}$​ is the characteristic cube strength of concrete in (kN). If the applied shear stress exceeds this value, shear reinforcement must be provided such that the reinforcement contribution is $\:\left({q}_{su}={q}_{u}-0.5{q}_{cu}\right)$. To avoid brittle diagonal cracking, the code also mandates a minimum amount of shear reinforcement, expressed as $\:\left(\frac{{A}_{sv}}{s}\ge\:\frac{0.35\:b}{{f}_{y}}\right)$.

Where $\:{A}_{sv}$​ is the cross-sectional area of stirrup legs in (mm^2^), s is the stirrup spacing in (mm), and $\:{f}_{y}\:$is the yield stress of the stirrup steel in (MPa). On the other hand, to prevent crushing of the web concrete, the maximum allowable shear stress is restricted to $\:{q}_{u\:max}=0.7*\:\sqrt{{f}_{\boldsymbol{c}\boldsymbol{u}\:}}$​​, not exceeding 4 N/mm². In conclusion, the Egyptian code restricts the maximum shear stress capacity of concrete and mandates a minimum degree of stirrup reinforcement in slender RC beams independent of the applied shear, guaranteeing both ductile characteristics and protection versus web crushing.

### Slender RC beams in ACI 318 − 19

To guarantee that slender beams can safely withstand shear stresses, the American Concrete Institute (ACI) provides exact equations for shear design (see ACI 318 − 19 Chap. 22 - Shear; Table 22.5.5.1 and related clauses (22.5.3 → 22.5.5). The key formulas and ideas that are used in the shear design process can be presented here as shown in Eq. 6. The shear strength ($\:{v}_{c}$) of the concrete in addition to the contribution ($\:{v}_{s}$) of the shear reinforcement add to the amount of a beam’s nominal shear strength, or$\:\:{v}_{n}\:$This can be stated as follows:

6 $$\:{v}_{n}={v}_{c}+\:{v}_{s}$$

#### Concrete contribution ($\:{\boldsymbol{v}}_{\boldsymbol{c}}$)

Applying Eq. 7, the concrete’s shear strength ($\:{v}_{c}$​) in (kN) was determined.

7 $$\:{v}_{c}=\varnothing\:*\:\sqrt{{f}_{\boldsymbol{c}\:}\boldsymbol{{\prime\:}}}*b*d$$

Where b is the beam’s width in (mm), d represents the effective depth in (mm),$\:\:{f}_{\boldsymbol{c}\:}^{\boldsymbol{{\prime\:}}}$ is the concrete’s compressive strength in (MPa), and ∅ is the strength reduction factor, which is equivalent to 0.75 for shear. The ACI produces precise values for ø based to the loading and condition of the beam.

#### Shear reinforcement contribution ($\:{\boldsymbol{V}}_{\boldsymbol{s}})$

Shear reinforcement contribution ($\:{v}_{s})$ was estimated by Eq. 8.

8 $$\:{v}_{s}=\frac{{A}_{v}*\:{f}_{y}*d*Sin\:\theta\:}{s}$$

Where $\:{A}_{v}$ is the shear reinforcement’s area in (mm^2^), $\:{f}_{y}\:$is the yield strength of the reinforcement in (MPa), d is the effective depth of the beam in (mm), θ is the stirrups angle of inclination (typically 45 degrees for vertical stirrups), and s is the spacing between stirrup in (mm).

ACI advises using minimum shear reinforcement if the predicted shear force is higher than the concrete’s stated shear strength. The ACI code specifies a minimum amount of shear reinforcement whenever the beam depth exceeds 250 mm or when the applied shear exceeds half the concrete shear strength.

#### The minimal shear reinforcement area

Equation 9 demonstrates that the minimal area of shear reinforcement is as follows.

9 $$\:{A}_{v\:min}=\frac{0.4*\:b\:*d}{{f}_{y}}$$

Where $\:{A}_{v\:min}$ is the minimum reinforcing requires in (mm^2^), b is the beam width in (mm), d is the beam effective depth in (mm), and $\:{f}_{y}$ is the yield strength of the reinforcement in (MPa), This minimum stirrup ratio is expressed as $\:\left(\frac{{A}_{v}}{s}>\frac{50\:b}{{f}_{y}}\right)$.

Where $$\:{A}_{v}$$​ is the area of stirrup legs within spacing s, b​ is the web width, and $$\:{f}_{y}$$​ is the yield strength of the stirrup steel. Additionally, the maximum spacing of stirrups is limited to the lesser of d/2–600 mm to ensure adequate crack control along the shear span. On the other end, maximum shear strength limits are enforced to prevent crushing of the concrete web, with the nominal shear strength restricted to ($$\:{v}_{n}\le\:0.66\sqrt{{f}_{\boldsymbol{c}\:}\boldsymbol{{\prime\:}}}*b*d)$$.

The nominal shear strength of slender beams is calculated by adding the contributions from shear reinforcement and concrete, according to ACI. Shear strength, minimum reinforcing requires, and stirrup spacing are all determined by specific equations. By adopting these recommendations, beams can efficiently withstand shear forces while preserving their overall structural integrity and serviceability as investigated by^22–25^. In conclusion, the ACI code provides balanced safety against both fractured and crushing failure modes by guaranteeing that slender beams always have a minimum amount of shear reinforcement to avert brittle diagonal cracking and limiting the ultimate shear capacity to avoid web crushing. ACI 318 − 19 requires a minimum tensile reinforcement ratio of 0.0018 (or 0.18%) of the gross cross-sectional area, rather than 0.2%.

### Slender RC beams in Eurocode 2 (EN 1992)

Eurocode 2 (EN 1992) establishes detailed standards for the shear design of slender reinforced concrete beams to guarantee structural integrity and safety. A “simplified formula” was derived from the European code (as given in Clause 6.2, standard notation) by approximating the original code limits (k) and the reinforcement effect using a linear ratio instead of a cube root. The following are the essential equations and principles pertinent to the shear design process.

#### Concrete contribution ($\:{\boldsymbol{V}}_{\boldsymbol{c}})$

Equation 10 provides the concrete’s contribution to a slender beam’s shear resistance ($\:{V}_{c})\:$without stirrups as observed by^26^.

10 $$\:{V}_{c}=\frac{0.0525}{{\gamma\:}_{c}}*{\left({f}_{c}^{{\prime\:}}\right)}^{\raisebox{1ex}{$2$}\!\left/\:\!\raisebox{-1ex}{$3$}\right.}*\left(1.6-d\right)\left(1.2+40{\rho\:}_{\lambda\:}\right)*b*d$$

Where γ_c​_ = 1.5 is the concrete material’s safety factor, $\:{f}_{c}^{{\prime\:}}$ is the concrete’s cylinder compression strength in (MPa), $\:{\rho\:}_{\lambda\:}$ is the longitudinal steel ratio, and $\:b$​ is the width of the web in (mm), $\:d$ reflects the web’s effective depth in (mm), respectively.

#### Shear reinforcement contribution ($\:{\boldsymbol{V}}_{\boldsymbol{s}})$

Equation (11) is used to evaluate the stirrups’ contribution to the shear capacity when shear reinforcement is utilized.

11 $$\:{V}_{s}={0.9*\rho\:}_{v}*{f}_{vy}*b*d$$

Where $\:{\rho\:}_{v}$.$\:\:{f}_{vy}$ is the stirrups’ nominal strength, b is the width of the beam in (mm), and d is its effective depth in (mm).

For the minimum, the code requires that a certain amount of shear reinforcement must always be provided to prevent brittle diagonal tension failure, even if the calculated shear demand is low. This minimum is defined ($\:\frac{{A}_{sw}}{s}\ge\:{\rho\:}_{w\:min}.\:{b}_{w}$) with.

$$\:({\rho\:}_{w\:min}=0.08\frac{\sqrt{{f}_{\boldsymbol{c}\boldsymbol{k}\:}}}{{f}_{yk}})$$

Where $\:{A}_{sw}$​ is the shear reinforcement within spacing s in (mm^2^), $\:{b}_{w}$​ is the web width in (mm), $\:{f}_{\boldsymbol{c}\boldsymbol{k}\:}\:$is the characteristic compressive strength of concrete in (MPa), and $\:{f}_{yk}$​ is the characteristic yield strength of the shear reinforcement in (MPa).

### Slender RC beams in CSA A23.3-1

The design shear strength of slender reinforced concrete (RC) beams in accordance with Canadian Standard CSA A23.3–19 is determined by the combined contribution of the concrete and the shear reinforcement. Equation 12 reveals the factored shear resistance $\:{V}_{r}$ CSA A23.3 (see shear sizing and reinforcement spacing, e.g., 11.3.8/related items depending on edition A23.3–14/A23.3–19).

12 $$\:{V}_{r}={V}_{c}+{V}_{s}$$

Where $\:{V}_{r}$ represents the section’s calculated shear resistance (kN), $\:{V}_{c}$ symbolizes the shear resistance of the concrete in (kN), and $\:{V}_{s}$ represents the shear resistance of the shear reinforcement in (kN).

#### Concrete contribution ($\:{\boldsymbol{V}}_{\boldsymbol{c}})$

For slender reinforced concrete beams, Eq. 13 demonstrates the concrete contribution of shear design (see CSA A23.3–14 (Eq. 11).

13 $$\:{v}_{c}={\varnothing\:}_{c}*\beta\:*\lambda\:*\:\sqrt{{f}_{\boldsymbol{c}\:}\boldsymbol{{\prime\:}}}*{b}_{w}*d$$

Where The resistance factor for concrete is $\:{\varnothing\:}_{c}$, which is normally 0.65, The empirical coefficient is signified by $\:\beta\:$, which is 0.18 for beams without axial force, $\:\lambda\:$ is the density factor of concrete, which is 1.0 for normal weight, $\:{f}_{\boldsymbol{c}\:}\boldsymbol{{\prime\:}}$ is the compressive strength of the concrete in (MPa), $\:{b}_{w}$ refers to the web beam width in (mm), and d refers to the effective depth in (mm).

#### Shear reinforcement contribution ($\:{\boldsymbol{V}}_{\boldsymbol{s}})$

Equation 14 shows the contribution of shear reinforcement to the shear design of slender reinforced concrete beams.

14 $$\:{v}_{s}={A}_{v}\mathrm{*}{f}_{yv}\mathrm{*}\frac{d}{s}\:\:\:\:\:\:\:\:\:\:\:\:$$

Where $\:{A}_{v}$ is the area of shear reinforcement (stirrups) in (mm^2^) per spacing s,$\:\:{f}_{yv}$ is the yield strength of the shear reinforcement in (MPa), d is the beam’s effective depth in (mm), and s is the spacing between stirrup in (mm).

#### Minimum shear reinforcement

The minimum stirrup area per unit length must meet the following requirements if shear reinforcement is needed. Equation 15 provides the minimum shear reinforcement that can be applied.

15 $$\:\frac{{A}_{v}}{s}\ge\:\frac{0.06\sqrt{{f}_{\boldsymbol{c}\:}\boldsymbol{{\prime\:}}}}{\:{f}_{y}}*{b}_{w}$$

The CSA determined the maximum limit of shear design as defined ($\:{v}_{max}={\propto\:}_{c}$.$\:z\:\mathrm{v}\:{b}_{w}{f}_{\boldsymbol{c}\:}\boldsymbol{{\prime\:}}$.$\:\:\frac{\mathrm{cot}\theta\:}{1+{\mathrm{cos}}^{2}\theta\:}$).

### Slender RC beams in BS 8110

The shear design is stated in BS 8110 (see Clause 3.4, 3.5 Equation numbers in the BS text). Estimating the nominal shear resistance of the concrete and evaluating if shear reinforcing needs to be provided based on applied shear resistance are steps in the shear design of RC slender beams. By following these recommendations, beams can successfully withstand shear stresses while preserving their structural soundness and usability as observed by^13,26^.

#### Concrete contribution ($\:{\boldsymbol{v}}_{\boldsymbol{c}}$)

For slender reinforced concrete beams, Eq. 16 shows the concrete contribution of shear design in accordance with BS 8110.

16 $$\:{v}_{c}=\frac{0.79}{{\gamma\:}_{m}}\:.\:{\left(\frac{100\:{A}_{s}}{{b}_{v}d}\right)}^{\raisebox{1ex}{$1$}\!\left/\:\!\raisebox{-1ex}{$3$}\right.}.{\left(\frac{400\:}{d}\right)}^{\raisebox{1ex}{$1$}\!\left/\:\!\raisebox{-1ex}{$4$}\right.}.{\left(\frac{\:{f}_{cu}}{25}\right)}^{\raisebox{1ex}{$1$}\!\left/\:\!\raisebox{-1ex}{$3$}\right.}.\mathrm{b}.\mathrm{d}\:$$

Where $\:{A}_{s}\:$is the longitudinal steel section in (mm^2^), $\:{b}_{v}$ is its width in (mm), d is its effective depth in (mm),$\:\:\:{f}_{cu}$ is its cube compression strength of concrete in (MPa) ($\:{f}_{c}$′ = 0,8 $\:{f}_{cu}$), and $\:{\gamma\:}_{m}$ is the material’s safety factor.

#### Shear reinforcement ​contribution ($\:{\boldsymbol{V}}_{\boldsymbol{s}})$

Equation 17 provides the shear reinforcement contribution of the shear design of slender reinforced concrete beams in accordance with BS 8110.

17 $$\:{v}_{s}=0.87{\rho\:}_{v}.\:{f}_{vy}.\mathrm{b}.\mathrm{d}\:$$

Where $\:{\rho\:}_{v}$.$\:\:{f}_{vy}$ is the nominal strength of stirrups in (MPa).

### Slender RC beams in Zsutty

Equations 18 and 19 can be used to compute the contribution of concrete and shear reinforcement according to^27,28^.

#### Concrete contribution ($\:{\boldsymbol{v}}_{\boldsymbol{c}}$)

18 $$\:{v}_{c}=2.2*{\left({\rho\:}_{\lambda\:}*{f}_{\boldsymbol{c}\:}^{\boldsymbol{{\prime\:}}}\boldsymbol{*}\frac{\mathrm{d}}{a}\right)}^{\raisebox{1ex}{$1$}\!\left/\:\!\raisebox{-1ex}{$3$}\right.}$$

#### Shear reinforcement ​contribution ($\:{\boldsymbol{V}}_{\boldsymbol{s}})$

19 $$\:{V}_{s}={\rho\:}_{v}*{f}_{vy}*b*d$$

Where a/d is the ratio of shear-span to depth, $\:{\rho\:}_{v}{f}_{vy}$ is stirrups’ nominal strength in (MPa), $\:d$ represents the beam’s effective depth in (mm), b is the beam’s width in (mm), $\:{\rho\:}_{\lambda\:}$ is the ratio of longitudinal steel, and $\:{f}_{\boldsymbol{c}\:}\boldsymbol{{\prime\:}}$ refers to the compressive strength of concrete in (MPa).

### Slender RC beams in JSCE

According to the JSCE, determining out the nominal shear resistance is required to ascertain slender beams’ shear capability (see JSCE equations in Chap. 9/Sect. 9.2 (shear/shear compression capacity Eqs. (20–24)).

#### Concrete contribution ($\:{\boldsymbol{v}}_{\boldsymbol{c}}$)

As stated on Eq. 20 of the concrete contribution, the concrete contributes and determines if shear reinforcing is necessary based on applied shear stresses.

20 $$\:{v}_{c}=\alpha\:*\beta\:*\sqrt{{f}_{\boldsymbol{c}\:}\boldsymbol{{\prime\:}}}*{b}_{w}*d$$

where $$\:{f}_{\boldsymbol{c}\:}\boldsymbol{{\prime\:}}$$ refers to the compressive strength of concrete in (MPa), $$\:{b}_{w}$$ is the width of the web in (mm), $$\:d$$ is the effective depth of the beam in (mm), $$\:{v}_{c}$$ is the shear strength of concrete in (kN) that concrete offers, $$\:\alpha\:$$ is the slenderness correction factor, and $$\:\beta\:$$ is the longitudinal reinforcement effect factor that can be computed using Eqs. 22 and 23. Equation 21 illustrates how JSCE reduces the shear capacity as the beam gets thinner by adding a slenderness correction factor (α), which is reliant on the ratio of span to depth ($$\:\raisebox{1ex}{$l$}\!\left/\:\!\raisebox{-1ex}{$d$}\right.$$).

21 $$\:\alpha\:=1-0.2*\left(\frac{\raisebox{1ex}{$l$}\!\left/\:\!\raisebox{-1ex}{$d$}\right.-2}{4}\right)\:\:\:\:for\:\:2<\raisebox{1ex}{$l$}\!\left/\:\!\raisebox{-1ex}{$d$}\right.\le\:6$$

22 $$\:\beta\:=1+2.5\rho\:$$

23 $$\:\rho\:=\frac{{A}_{s}}{{b}_{w}*d}\:\left(\mathrm{l}\mathrm{o}\mathrm{n}\mathrm{g}\mathrm{i}\mathrm{t}\mathrm{u}\mathrm{d}\mathrm{i}\mathrm{n}\mathrm{a}\mathrm{l}\:\mathrm{r}\mathrm{e}\mathrm{i}\mathrm{n}\mathrm{f}\mathrm{o}\mathrm{r}\mathrm{c}\mathrm{e}\mathrm{m}\mathrm{e}\mathrm{n}\mathrm{t}\:\mathrm{r}\mathrm{a}\mathrm{t}\mathrm{i}\mathrm{o}\right)\:$$

#### Shear reinforcement ​contribution ($\:{\boldsymbol{V}}_{\boldsymbol{s}})$

In the event that the design shear force surpasses the concrete’s nominal shear capacity, shear reinforcement () in (kN) is required. Equation 24 provides the contribution from shear reinforcement.

24 $$\:{v}_{s}=\frac{{A}_{sv}*\:{f}_{y}*d}{s}$$

The shear reinforcement area is denoted by $\:{A}_{sv}$ in (mm^2^), $\:{f}_{y}$ is the stirrup yield strength in (MPa), and $\:s$ is the stirrup spacing in (mm).

A minimum amount of shear reinforcement must always be provided, regardless of the calculated shear demand, in order to maintain post-cracking ductility and avoid sudden diagonal tension failure. This requirement is expressed as $\:\frac{{A}_{v}}{{b}_{w}*s}\ge\:0.002$ Where $\:{A}_{v}\:$is the area of shear reinforcement within spacing s, and ​ $\:{b}_{w}\:$is the beam web width. On the other hand, the maximum shear strength is restricted by the crushing capacity of the concrete struts in the web. JSCE limits the design shear resistance to (V_max_ $\:\le\:0.25\:{f}_{c\:}{\prime\:}{b}_{w}*d$) Where $\:{f}_{c\:\:}^{{\prime\:}}$is the design compressive strength of concrete in (MPa), $\:{b}_{w}$ is the web width in (mm), and d is the effective depth in (mm). These provisions ensure that reinforced concrete beams are provided with a safe amount of shear reinforcement while avoiding unsafe diagonal compression failure of the web.

## Shear design for non slender beams

### Non slender RC beams in ECP 203–2020

#### Nominal shear strength provided by concrete

For beams subjected to shear (without torsion), the ultimate shear strength of concrete ($\:{q}_{cu}$) is calculated by Eq. 25.

25 $$\:{q}_{cu}=0.24*\:\sqrt{{f}_{cu}}$$

#### Maximum allowable shear stress

The nominal shear stress $\:{q}_{u\:}$​ must not exceed:

26 $$\:{q}_{u\:max}=0.7*\:\sqrt{{f}_{cu}}\le\:4\:N/{mm}^{2}$$

#### Shear reinforcement requirement

If the applied shear stress $\:{q}_{u\:}$​ exceeds the concrete’s shear capacity $\:{q}_{cu\:}$, shear reinforcement must be provided such that:

27 $$\:\mathrm{I}\mathrm{f}\:{(q}_{u}>{q}_{cu})\:{q}_{su}=\:{q}_{u}-0.5{q}_{cu}$$

Where $\:{q}_{su}$ is the portion resisted by reinforcement.

### Non slender RC beams in ACI 318 − 19

In ACI 318, the shear design of non-slender reinforced concrete (RC) beams involves several key formulas and guidelines to ensure adequate shear strength. Here are the main components, along with maximum and minimum limits.

#### Nominal shear strength ($\:{\boldsymbol{v}}_{\boldsymbol{n}}$​)

The nominal shear strength of a non-slender RC beam is given by Eq. 28.

28 $$\:{V}_{n}={V}_{c}+{V}_{s}$$

Where $\:{V}_{n}$ is the nominal shear strength in (kN), ​$\:{V}_{n}$ is the Shear strength provided by the concrete (kN) and $\:{V}_{s}$ is the shear strength provided by the shear reinforcement (kN).

#### Concrete contribution ($\:{\boldsymbol{V}}_{\boldsymbol{c}}$​)

The shear strength provided by the concrete is calculated using Eq. 29.

29 $$\:{V}_{c}=0.17\sqrt{{f}_{\boldsymbol{c}\:}\boldsymbol{{\prime\:}}}*b*d$$

Where b is the beam’s width in (mm), d is the effective depth in (mm), and$\:\:{f}_{\boldsymbol{c}\:}^{\boldsymbol{{\prime\:}}}$ is the concrete’s compressive strength in (MPa).

#### Shear reinforcement contribution ($\:{\boldsymbol{V}}_{\boldsymbol{s}}$​)

The contribution of shear reinforcement is calculated by Eq. 30.

30 $$\:{v}_{s}=\frac{{A}_{v}*\:{f}_{y}*{l}_{v}}{s}$$

Where $\:{A}_{v}$ is the area of shear reinforcement in (mm²), $\:{f}_{y}$ is the yield strength of the shear reinforcement in (MPa), $\:{l}_{v}$ is the length of the vertical leg of the stirrups in (mm), and s is the spacing of the shear reinforcement in (mm).

#### Minimum shear reinforcement

According to ACI 318, the minimum shear reinforcement is required to prevent shear failure and is given by Eq. 31.

31 $$\:{A}_{v\:min}=\frac{0.4*\:b\:*{f}_{\boldsymbol{c}\:}\boldsymbol{{\prime\:}}}{{f}_{y}}$$

Where b is the width of the beam in (mm), $\:{f}_{\boldsymbol{c}\:}\boldsymbol{{\prime\:}}$​ is the specified compressive strength of concrete in (MPa), and $\:{f}_{y}$​ is the yield strength of the shear reinforcement in (MPa).

#### Maximum shear reinforcement spacing

The maximum spacing of shear reinforcement is limited to ensure effective shear resistance. ACI 318 specifies:

$\:{s}_{max}$= the minimum of ($\:\frac{d}{2}$ ​, 450 mm, 600 mm).

### Non slender RC beams in EN 92

The shear design of non-slender reinforced concrete (RC) beams in Eurocode 2 (EN 1992) follows specified regulations and criteria. The formulas and standards from Eurocode 2 (EN 1992) offer an extensive basis for the shear design of non-slender concrete- reinforced beams, guaranteeing sufficient safety and performance under diverse loading circumstances.

#### Nominal shear strength provided by concrete

A non-slender RC beam’s overall design shear resistance can be represented from Eq. 32.

32 $$\:{V}_{R}={V}_{c}+{V}_{s}$$

Where $\:{V}_{R}$ is the design shear force at the section in (kN), $\:{V}_{R}$ is the shear strength provided by concrete in (kN), and $\:{V}_{s}$ is the shear strength provided by shear reinforcement (kN).

#### Concrete contribution ($\:{\boldsymbol{V}}_{\boldsymbol{c}}$​)

The following formula 33 is used to determine the concrete’s shear strength.

33 $$\:V_{c} = \frac{{0.12*\sqrt {f_{{ck}} } }}{{\gamma \:_{c} }}*b*d~(for\:f_{{ck}} \: = 30MPa)$$

Where b is the width of the beam in (mm), $\:d$ is the effective depth of the beam in (mm), and γ_c_​ is the partial safety factor for concrete (typically taken as 1.5).

#### Shear reinforcement contribution ($\:{\boldsymbol{V}}_{\boldsymbol{s}}$​)

The contribution from shear reinforcement is calculated from Eq. 34.

34 $$\:Vs=\frac{{A}_{sv}*{f}_{yv}*{l}_{v}}{s}$$

Where $\:{A}_{sv}$ is the area of shear reinforcement in (mm²), $\:{f}_{yv}$ is the yield strength of the shear reinforcement in (MPa), $\:{l}_{v}$ is the length of the shear reinforcement in (mm) (typically the vertical leg of stirrups), and s is the spacing of the shear reinforcement in (mm).

#### Minimum shear reinforcement

The minimum shear reinforcement is required to ensure safety against shear failure can be calculated from Eq. 35.

35 $$\:{A}_{sv\:min}=0.4*\frac{b*{f}_{ck}}{{f}_{yv}}$$

#### Maximum shear reinforcement spacing

The maximum spacing of shear reinforcement must not exceed:

$$s_{{\max }} = the\min imum\,of\,(\frac{d}{2}\,,\,450mm)$$

### Non slender RC beams in CSA

The shear design for non-slender reinforced concrete (RC) beams is specified in the Canadian Standards Association (CSA) standard for concrete structure design, encompassing certain formulas and instructions. This is a concise overview of the shear design procedure for non-slender beams, as specified by the CSA.

#### Nominal shear strength provided by concrete (V_u_)

The total shear force acting on a non-slender RC beam is expressed at 36.

36 $$\:{V}_{u}={V}_{c}+{V}_{s}$$

Where $\:{V}_{u}$ is the design shear force at the section (kN), $\:{V}_{c}$ is the shear strength provided by concrete (kN) and $\:{V}_{s}$ is the shear strength provided by shear reinforcement (kN).

#### Concrete contribution (V_c_)

The shear strength provided by the concrete is given by Eq. 37.

37 $$\:{V}_{c}=0.25\sqrt{{f}_{\boldsymbol{c}\:}}*b*d$$

Where $\:{f}_{\boldsymbol{c}\:}$is the compressive strength of concrete, b is the width of the beam (mm) and d is the effective depth of the beam (mm).

#### Shear reinforcement contribution (V_s_)

The contribution from shear reinforcement can be calculated by Eq. 38.

38 $$\:{V}_{s}={A}_{v}*{f}_{y}*\frac{d}{s}$$

Where $\:{A}_{v}$ is the area of shear reinforcement (mm²), $\:{f}_{y}$ is the yield strength of the shear reinforcement (MPa), and s is the spacing of the shear reinforcement (mm).

#### Minimum shear reinforcement

The minimum shear reinforcement is required to ensure safety against shear failure and is calculated as shown in Eq. 39.

39 $$\:{A}_{v\:min}=0.4*\frac{b*{f}_{c}}{{f}_{y}}$$

#### Maximum shear reinforcement spacing

The maximum spacing of shear reinforcement should not exceed $\:{S}_{max}=\mathrm{min}of\:(\frac{d}{2}.\:450\:mm)$.

### Non slender RC beams in BS 8110

The British Standard BS 8110 delineates the shear design for non-slender reinforced concrete beams, using specific formulas for concrete structure design.

#### Total design shear force

The total design shear force acting on a non-slender RC beam is expressed as shown in Eq. 40.

40 $$\:{V}_{u}={V}_{c}+{V}_{s}$$

#### Concrete contribution $\:\left({\boldsymbol{V}}_{\boldsymbol{c}}\right)$

The shear strength provided by the concrete is calculated using Eq. 41.

41 $$\:{v}_{c}=\left(2*\frac{d}{a}\right)\mathrm{*}\frac{0.79}{{\gamma\:}_{m}}\:\mathrm{*}{\left(\frac{100\:{A}_{s}}{{b}_{v}d}\right)}^{\raisebox{1ex}{$1$}\!\left/\:\!\raisebox{-1ex}{$3$}\right.}\mathrm{*}{\left(\frac{400\:}{d}\right)}^{\raisebox{1ex}{$1$}\!\left/\:\!\raisebox{-1ex}{$4$}\right.}\mathrm{*}{\left(\frac{\:{f}_{cu}}{25}\right)}^{\raisebox{1ex}{$1$}\!\left/\:\!\raisebox{-1ex}{$3$}\right.}\mathrm{*}\mathrm{b}\mathrm{*}\mathrm{d}\:$$

#### Shear reinforcement contribution $\:\left({\boldsymbol{V}}_{\boldsymbol{s}}\right)$

The contribution from shear reinforcement can be determined as shown in Eq. 42.

42 $$\:{V}_{s}=\frac{{A}_{v}*{f}_{y}*{l}_{v}}{s}$$

Where $\:{A}_{v}$​ is the area of shear reinforcement (mm²), $\:{f}_{y}$ is the yield strength of the shear reinforcement (MPa), $\:{l}_{v}\:$is the length of the shear reinforcement (typically the vertical leg of stirrups), and s is the spacing of the shear reinforcement (mm).

#### Minimum shear reinforcement

The minimum shear reinforcement is specified to ensure adequate resistance to shear as shown in Eq. 43.

43 $$\:{A}_{v\:min}=0.4*\frac{b*{f}_{cu}}{{f}_{y}}$$

#### Maximum shear reinforcement spacing

The maximum spacing of shear reinforcement should not exceed $\:{S}_{max}=\mathrm{min}of\:(\:\frac{d}{2}.\:300\:mm)$.

### Non slender RC beams in JSCE

The shear design procedure for non-slender (deep) reinforced concrete beams, as delineated by the Japan Society of Civil Engineers (JSCE), markedly contrasts with that for slender beams. According to JSCE rules, slender beams are characterized by a span-to-depth ratio (l/d) of 2 or above, whereas non-slender beams possess a span- to-depth ratio of less than 2. This distinction is crucial because, in non-slender beams, flexural theory is less applicable; instead, shear transfer primarily occurs through arching action rather than traditional beam action. The shear strength for non-slender beams is expressed as shown in Eq. 44.

44 $$\:{V}_{u}={V}_{cd}+{V}_{sd}$$

Where $\:{V}_{cd}$ represents the shear resistance of the concrete, which is based on compressive strut action and arching, and $\:{V}_{sd}$​ denotes the shear resistance provided by shear reinforcement, such as stirrups or welded mesh.

#### Nominal shear strength provided by concrete

For non-slender beams, the concrete contribution to shear resistance is given by Eq. 45.

45 $$\:{V}_{cd}={\upalpha\:}\mathrm{*}{b}_{w}*{j}_{d}*{f}_{t}$$

Where α is a coefficient that decreases as the beam becomes deeper (typically around 1.0 for deep beams exhibiting strong arching), b_w_​ is the web width in (mm), j_d_​ (approximately 0.9d) is the lever arm, and f_t_​ is the tensile strength of the concrete in (MPa), calculated as 0.56fc′ (MPa).

#### Shear reinforcement contribution ($\:{\boldsymbol{V}}_{\boldsymbol{s}\boldsymbol{d}})$

The shear reinforcement contribution is defined as shown in Eq. 46.

46 $$\:V_{{sd}} = \frac{{A_{{sv}} *f_{{yv}} *z}}{s} \cdot \cot \theta \:$$

Where $\:{A}_{sv}$ is the area of shear reinforcement per spacing s (mm^2^), $\:{f}_{yv}$ is the yield strength of stirrups in (MPa), $\:z$ = 0.9.d, and θ = angle of diagonal compression strut (often taken as 45° for deep beams).

## Solved example

Beam Sect. (300 * 600)

### Shear design according to ECP-2020

*Given data for Rectangular beam section*

b = 300 mm, d = 550 mm, $\:{f}_{\boldsymbol{c}\boldsymbol{u}\:}$**=** 30 MPa, $\:{\uptheta\:}$ = 45°, $\:\mathrm{c}\mathrm{o}\mathrm{t}\left({\uptheta\:}\right)$ = 1, (2 leg stirrups Ø 8 or 2 leg stirrups Ø 10 spaced at 150 mm), $\:{f}_{\boldsymbol{y}\:\boldsymbol{s}\boldsymbol{t}\boldsymbol{r}\:}$**=** 240 MPa.

#### Solution

1. Concrete contribution

$$\:{v}_{c}=0.16*\:\sqrt{{f}_{\boldsymbol{c}\boldsymbol{u}\:}}*b*d*{10}^{-3}$$

$$\:{v}_{c}=0.16*\:\sqrt{30}*300*550*{10}^{-3}$$ = 144.5 kN.

2. Shear reinforcement contribution

$$\:{v}_{s}=\frac{{A}_{sv}*{f}_{y}*d\:}{s}*\mathrm{c}\mathrm{o}\mathrm{t}\left({\uptheta\:}\right)\mathrm{*}{10}^{-3}$$

If Ø 8 single leg $\:{A}_{sv}$ = 50.265 mm^2^, Ø 10 single leg $\:{A}_{sv}$ = 78.54 mm^2^.

For 2 leg Ø 8 $\:{A}_{sv}$ = 100.531 mm^2^, with s = 150 mm.

$$\:{v}_{s}\:=\frac{100.531\:*240*550\:}{150}*1\mathrm{*}{10}^{-3}$$= 88.46 kN.

2 leg Ø 10 $\:{A}_{sv}$ = 157.08 mm^2^, with s = 150 mm.

$$\:{v}_{s}\:=\frac{157.08\:*240*550\:}{150}*1\mathrm{*}{10}^{-3}$$= 138.23 kN.

3. Total shear strength

$$\:{v}_{u}={v}_{c}+\:{v}_{s}$$ = 144.5 + 88.46 = 233.006 kN (for Ø 8).

$$\:{v}_{u}={v}_{c}+\:{v}_{s}$$ = 144.5 + 138.23 = 282.8 kN (for Ø 10).

4. Minimum shear reinforcement

$$\:\left(\frac{{A}_{sv}}{s}\ge\:\frac{0.35\:b}{{f}_{y}}\right)$$

$$\:\frac{0.35\:b}{{f}_{y}}$$ = $$\:\frac{0.35*300}{240}$$ = 0.4375 mm^2^/mm.

$\:\frac{{A}_{sv}}{s}$ = $\:\frac{100.53}{150}$ = 0.6702 mm^2^/mm ((for Ø 8) (ok).

$\:\frac{{A}_{sv}}{s}$ = $\:\frac{157.08\:}{150}$ = 1.047 mm^2^/mm ((for Ø 10) (ok).

5. Maximum shear reinforcement

$$\:{q}_{u\:max}=0.7*\:\sqrt{{f}_{\boldsymbol{c}\boldsymbol{u}\:}}$$ =$$\:0.7*\:\sqrt{30}$$ = 3.83 MPa

$$\:{v}_{\:max}={q}_{u\:max}*\:b*d*{10}^{-3}$$

= 3.83 * 165,000* $$\:{10}^{-3}$$ = 632.618 kN.

$\:{v}_{\:u}$ = 283 kN ˂˂ $\:{v}_{\:max}$ = 632.618 kN (ok).

### Shear design according to ACI 318 − 19

*Given data for Rectangular beam section*.

b = 300 mm, t = 600 mm, d = 550 mm, (2 leg stirrups Ø 8 at 150 mm spacing), $\:{f}_{\boldsymbol{y}\:}$**=** 460 MPa, $\:{f}_{\boldsymbol{c}\:}\boldsymbol{{\prime\:}}$**=** 30 MPa, $\:\varnothing\:$ = 0.75.

#### Solution

Assume = 90°.

1. Concrete contribution

$$\:{v}_{c}=\varnothing\:*\:\sqrt{{f}_{\boldsymbol{c}\:}\boldsymbol{{\prime\:}}}*b*d$$ = 0.75*5.44*300*550 = 678 kN.

2. Shear reinforcement contribution.

$$\:{v}_{s}=\frac{{A}_{v}*\:{f}_{y}*d*Sin\:\theta\:}{s}$$ = $$\:\frac{100.5*460*550*1}{150}$$ = 169 kN.

3. Nominal shear capacity

$$\:{v}_{n}={v}_{c}+\:{v}_{s}$$ = 678 + 169 = 847 kN.

4. Minimum shear reinforcement

$$\:\left(\frac{{A}_{v}}{s}>\frac{50\:b}{{f}_{y}}\right)$$

$$\:\frac{{A}_{v}}{s}$$ = $$\:\frac{100.5}{150}$$ = 0.67 mm^2^/mm.

$$\:\frac{50\:b}{{f}_{y}}$$ = $$\:\frac{50*300}{460}$$ = 32.6 mm^2^/mm.

0.67 mm^2^/mm ˂˂ 32.6 mm^2^/mm (Minimum reinforcement is not satisfied).

$\:\frac{{A}_{v}}{s}$ = 32.6*150 = 4890 mm^2^ (More than 2 Ø 8 at 150 mm).

ACI minimum shear requirements is very demanding compared to BS/EC2)

5. Maximum shear strength

($$\:{v}_{n}\le\:0.66\sqrt{{f}_{\boldsymbol{c}\:}\boldsymbol{{\prime\:}}}*b*d)$$

$$\:{v}_{u\:max}=0.66*\sqrt{30}*300*550$$ = 595 kN.

$$\:{v}_{n}$$ = 847 kN ˃ $$\:{v}_{u\:max}$$= 595 kN.

(The design must be reduced to 595 kN)

### Shear design according to EN 92

*Given data for Rectangular beam section*.

b = 300 mm, t = 600 mm, d = 550 mm, $\:{f}_{\boldsymbol{c}\:}\boldsymbol{{\prime\:}}$**=** 30 MPa, $\:{f}_{\boldsymbol{v}\boldsymbol{y}\:}$**=** 250 MPa, $\:{\rho\:}_{\lambda\:}$**=** 0.015, $\:\rho _{v}$**=** 0.002, $\:{f}_{yk}=500\:\mathrm{M}\mathrm{P}\mathrm{a}$

#### Solution

1. Concrete contribution

$$\:{V}_{c}=\frac{0.0525}{{\gamma\:}_{c}}*{\left({f}_{c}^{{\prime\:}}\right)}^{\raisebox{1ex}{$2$}\!\left/\:\!\raisebox{-1ex}{$3$}\right.}*\left(1.6-d\right)\left(1.2+40{\rho\:}_{\lambda\:}\right)*b*d$$

$$\:{V}_{c}=\frac{0.0525}{1.5}*{\left(30\right)}^{\raisebox{1ex}{$2$}\!\left/\:\!\raisebox{-1ex}{$3$}\right.}*\left(1.6-0.55\right)\left(1.2+40*0.015\right)*0.3*0.55$$ = 110 kN.

2. Shear reinforcement contribution

$$\:{V}_{s}={0.9*\rho\:}_{v}*{f}_{vy}*b*d$$

$$\:{V}_{s}=0.9*0.002*250*300*550$$ = 74.3 kN.

3. Total shear strength

$$\:{v}_{n}={v}_{c}+\:{v}_{s}$$ = 110 + 74.3 = 184.3 kN.

4. Minimum shear reinforcement

$$\:\frac{{A}_{sw}}{s}\ge\:{\rho\:}_{w\:min}*b$$

$$\:{\rho\:}_{w\:min}=0.08*\frac{\sqrt{{f}_{ck}}}{{f}_{yk}}$$ = 0.08*$$\:\frac{\sqrt{30}}{500}$$ = 8.76*10^− 4^.

$$\:{\rho\:}_{w\:min}*b$$ = 0.000876 * 300 = 0.263 mm^2^/mm.

$$\:\frac{{A}_{sw}}{s}$$ = $$\:\frac{100.53}{150}$$ = 0.6702 mm^2^/mm.

$\:\frac{{A}_{sw}}{s}=0.6702\ge\:{\rho\:}_{w\:min}*b=\:$ 0.263 mm^2^/mm ok.

5. Maximum shear reinforcement

$$\:\mathrm{cot}\theta\:=2.5\:,\:v=0.6\:(1-\frac{{f}_{ck}}{250}\:)\:=0.528$$

α_c_*​b_w_*​z*v*f_cd_​=1.0 × 300 × 495 × 0.528 × 20 = 1,568,160 N.

$$\:\mathrm{cot}\theta\:+\mathrm{tan}\theta\:$$ =2.5 + 0.4 = 2.9.

$$\:{v}_{u\:max}=\frac{1568160}{2.9}$$ = 541 kN.

$\:{v}_{n}=$184.3 kN ˂˂ $\:{v}_{u\:max}=\:$541 kN ok.

### Shear design according to CSA

*Given data for Rectangular beam section*.

b_w_ = 300 mm, t = 600 mm, d = 550 mm, $\:{f}_{\boldsymbol{c}\:}\boldsymbol{{\prime\:}}$**=** 30 MPa, $\:{\uplambda\:}=1$, Ø_c_ = 0.18, ꞵ = 1+$\:\sqrt{\frac{200}{d}}$ = 1+$\:\sqrt{\frac{200}{550}}$ = 1.603, $\:{f}_{yv}=400MPa$

assume 2 leg stirrups Ø 8 at 150 mm spacing.

$$\:{A}_{v}=\:\frac{2*\pi\:*{d}^{2}}{4}\:=\:100.53\:\mathrm{m}\mathrm{m}$$^2^.

ν = 0.6 (1- $$\:\frac{{f}_{ck}}{250}$$) = 0.528.

z = 0.9 d.

#### Solution

1. Concrete contribution

$$\:{v}_{c}={\varnothing\:}_{c}*\beta\:*\lambda\:*\:\sqrt{{f}_{\boldsymbol{c}\:}\boldsymbol{{\prime\:}}}*{b}_{w}*d$$

$$\:{v}_{c}=0.18*1.603*1*\:\sqrt{30}*300*550$$ = 260.8 kN.

2. Shear reinforcement contribution

$$\:{v}_{s}={A}_{v}\mathrm{*}{f}_{yv}\mathrm{*}\frac{d}{s}\:\:=\:100.53\mathrm{*}400\mathrm{*}\frac{550}{150}\:=\:147.4\:\mathrm{k}\mathrm{N}$$

3. Total shear strength

$$\:{v}_{r}={v}_{c}+\:{v}_{s}$$ = 260.8 + 147.4 = 408.2 kN.

4. Minimum shear reinforcement.

$$\:\frac{{A}_{v}}{s}=\frac{100.53}{150}=\frac{0.67{\mathrm{m}\mathrm{m}}^{2}}{\mathrm{m}\mathrm{m}}\ge\:\frac{0.06\sqrt{{f}_{\boldsymbol{c}\:}\boldsymbol{{\prime\:}}}}{\:{f}_{y}}*{b}_{w}=\:0.246{\mathrm{m}\mathrm{m}}^{2}/\mathrm{m}\mathrm{m}\:$$ ok.

5. Maximum shear strength

$$\:{v}_{max}={\propto\:}_{c}.z\:\mathrm{v}\:{b}_{w}{f}_{\boldsymbol{c}\:}\boldsymbol{{\prime\:}}.\:\frac{\mathrm{cot}\theta\:}{1+{\mathrm{cos}}^{2}\theta\:}$$ $$\:{v}_{max}=1\mathrm{*}495*\:0.528\mathrm{*}300\mathrm{*}30*\frac{2.5}{1+{2.5}^{2}}\:=\:811.1\:\mathrm{k}\mathrm{N}$$

$$v_{m} ax = 1*495*0.528*300*30*2.5/(1 + 2.5^{2} ) = 811.1kN$$

$\:{v}_{r}=$ 408.2 kN ˂ $\:{v}_{max}=$ 811.1 kN ok.

### Shear design according to BS 8110

*Given data for Rectangular beam section*.

b_v_ = 300 mm, t = 600 mm, d = 550 mm, $\:{f}_{\boldsymbol{c}\boldsymbol{u}\:}$ = 30 MPa,

$\:{\rho\:}_{l}$**=** 0.015, $\:{\gamma\:}_{m}=1.25$, 2 leg stirrups Ø 8 at 150 mm spacing, $\:{f}_{vy}$= 460 MPa.

A_s_= $$\:{\rho\:}_{l}*{b}_{v}*d$$= 0.015*300*550 = 2475 mm^2^.

#### Solution

1. Concrete contribution

$$\:{v}_{c}=\frac{0.79}{{\gamma\:}_{m}}\:.\:{\left(\frac{100\:{A}_{s}}{{b}_{v}d}\right)}^{\raisebox{1ex}{$1$}\!\left/\:\!\raisebox{-1ex}{$3$}\right.}.{\left(\frac{400\:}{d}\right)}^{\raisebox{1ex}{$1$}\!\left/\:\!\raisebox{-1ex}{$4$}\right.}.{\left(\frac{\:{f}_{cu}}{25}\right)}^{\raisebox{1ex}{$1$}\!\left/\:\!\raisebox{-1ex}{$3$}\right.}.\mathrm{b}.\mathrm{d}$$

$$\:\frac{100\:{A}_{s}}{{b}_{v}d}\:=\:100\mathrm{*}\frac{2475}{165000}=1.5*({1.5)}^{\raisebox{1ex}{$1$}\!\left/\:\!\raisebox{-1ex}{$3$}\right.}\:=\:1.145$$

$$\:{\left(\frac{400\:}{d}\right)}^{\raisebox{1ex}{$1$}\!\left/\:\!\raisebox{-1ex}{$4$}\right.}\:={\left(\frac{400\:}{550}\right)}^{\raisebox{1ex}{$1$}\!\left/\:\!\raisebox{-1ex}{$4$}\right.}\:=\:0.923$$

$$\:{\left(\frac{\:{f}_{cu}}{25}\right)}^{\raisebox{1ex}{$1$}\!\left/\:\!\raisebox{-1ex}{$3$}\right.}\:=\:{\left(\frac{\:30}{25}\right)}^{\raisebox{1ex}{$1$}\!\left/\:\!\raisebox{-1ex}{$3$}\right.}\:=\:1.062$$

$$\:\frac{0.79}{{\gamma\:}_{m}}\:=\:\frac{0.79}{1.25}\:=0.632$$

b.d = 165,000 mm^2^.

$$\:{v}_{c}=\:$$0.632*1.145*0.923*1.062*165,000 = 117kN.

2. Shear reinforcement contribution

$$\:{v}_{s}=0.87{\rho\:}_{v}.\:{f}_{vy}.\mathrm{b}.\mathrm{d}$$

For 2 Ø 8 $$\:{A}_{sv}$$ =2* $$\:\frac{\pi\:*{8}^{2}}{4}$$ = 100.53 mm^2^

$$\:{\rho\:}_{v}\:=\:\frac{100.53}{300*150}\:=\:0.002234$$

$$\:{v}_{s}=0.87{\rho\:}_{v}.\:{f}_{vy}.\mathrm{b}.\mathrm{d}$$

= 0.87* 0.002234*460*165,000 = 148 kN.

3. Total shear strength

$$\:{v}_{r}={v}_{c}+\:{v}_{s}$$ = 117 + 148 = 265 kN.

4. Minimum shear reinforcement

$$\:\frac{\:{A}_{sv}}{{b}_{v}s}=0.002234\:\ge\:\:\frac{\:0.4}{{f}_{yv}}=\frac{\:0.4}{460}=0.000869$$ok.

5. Maximum shear strength

$$\:{v}_{max}={\tau\:}_{c\:max}$$.b.d.

$\:{\tau\:}_{c\:max}$= 3.5 N/mm^2^ (from Tables 3, 4, 5, 6, 7 and 8).

$$\:{v}_{max}=3.5$$*165,000 = 577.5 kN.

$\:{v}_{r}=$265 kN ˂ $\:{v}_{max}=\:$577.5 kN ok.

### Shear design according to JSCE

*Given data for Rectangular beam section*.

b_w_ = 300 mm, t = 600 mm, d = 550 mm, $\:{f}_{\boldsymbol{c}\:}\boldsymbol{{\prime\:}}$**=** 30 MPa,

$$\:{\rho\:}_{l}$$**=** 0.015, assume l/d = 5, $$\:{f}_{\boldsymbol{y}\:}$$**=** 360 MPa.

ꞵ = 1 + 2.5⍴ =1 + 2.5(0.015) = 1.0375

$$\:\alpha\:=1-0.2*\frac{(\frac{l}{d}-2)}{4}\:=\:1-0.2*\frac{(5-2)}{4}\:=0.85$$

Assume 2 leg Ø 10 at 150 mm spacing.

$$\:{A}_{b}$$= 78.5 mm^2^.

2 leg $$\:{A}_{sv}$$= 157 mm^2^.

1. Concrete contribution

$$\:{v}_{c}=\alpha\:*\beta\:*\sqrt{{f}_{\boldsymbol{c}\:}\boldsymbol{{\prime\:}}}*{b}_{w}*d$$

= 0.85*1.0375*5.477*165,000 = 798 kN.

2. Shear reinforcement contribution

$$\:{v}_{s}=\frac{{A}_{sv}*\:{f}_{y}*d}{s}=\:\frac{157*\:360*550}{150}=\:\:207\mathrm{k}\mathrm{N}\:\:\:\:\:\:$$

3. Total shear resistance

$$\:{v}_{T}={v}_{c}+\:{v}_{s}$$ = 798 + 207 = 1005 kN.

4. Minimum shear reinforcement

$$\:\frac{{A}_{v}}{{b}_{w}*s}\ge\:0.002$$

0.002*$$\:{b}_{w}$$ = 0.6 mm^2^/mm.

Assume 2 leg Ø 8 at 150 mm spacing $\:{A}_{v}$= 100.5 mm^2^

5. Maximum shear strength

$$\:{v}_{max}\:=0.25\:{f}_{\boldsymbol{c}\:}\boldsymbol{{\prime\:}}{b}_{w}*d$$

= 0.25*30*300*550 = 1237.5kN.

$$\:{v}_{T}=$$ 1005 kN ˂ $$\:{v}_{max}$$=1237.5kN ok.

Beam Sect. (50 * 200).

### Shear design according to ECP

*Given data for Rectangular beam section*.

b = 50 mm, d = 180 mm, $\:{f}_{cu}$= 30 MPa, $\:{f}_{y\:str}$= 240 MPa, $\:\mathrm{cot}\theta\:=1$, $\:\theta\:$=45°,

#### Solution

1. Concrete contribution

$$\:{v}_{c}=0.16*\:\sqrt{{f}_{\boldsymbol{c}\boldsymbol{u}\:}}*b*d*{10}^{-3}$$

$$\:{v}_{c}=0.16*\:\sqrt{30}*50*180*{10}^{-3}$$ = 7.887 kN.

2. Shear reinforcement contribution

$$\:{v}_{s}=\frac{{A}_{sv}*{f}_{y}*d\:}{s}*\mathrm{c}\mathrm{o}\mathrm{t}\left({\uptheta\:}\right)\mathrm{*}{10}^{-3}$$

Case [A] Ø 8 single leg spaced at 150 mm.

$$\:\frac{{A}_{sv}}{s}$$ = $$\:\frac{50.27}{150}$$ = 0.335 mm^2^/mm.

$$\:{v}_{s}=\frac{50.27*240*180\:}{150}*{10}^{-3}= 14.476 kN$$

 

$$\:{v}_{u}={v}_{c}+\:{v}_{s}$$ = 7.887 + 14.476 = 22.36 kN.

Case [B] Ø 10 single leg spaced at 150 mm.

$$\:\frac{{A}_{sv}}{s}$$ = $$\:\frac{78.54}{150}$$ = 0.523 mm^2^/mm.

$$\:{v}_{s}=\frac{78.54*240*180\:}{150}*{10}^{-3}= 22.619 kN$$

 

$$\:{v}_{u}={v}_{c}+\:{v}_{s}$$ = 7.887 + 22.619 = 30.506 kN.

3. Minimum shear reinforcement

$$\:\left(\frac{{A}_{sv}}{s}\ge\:\frac{0.35\:b}{{f}_{y}}\right)$$

$$\:\frac{0.35\:b}{{f}_{y}}$$ = $$\:\frac{0.35*50}{240}$$ = 0.0729 mm^2^/mm.

$\:\frac{{A}_{sv}}{s}$ = $\:\frac{50.27}{150}$ = 0.335 mm^2^/mm (ok).

4-Maximum shear reinforcement.

$$\:{q}_{u\:max}=0.7*\:\sqrt{{f}_{\boldsymbol{c}\boldsymbol{u}\:}}$$ =$$\:0.7*\:\sqrt{30}$$ = 3.83 MPa

$$\:{v}_{\:max}={q}_{u\:max}*\:b*d*{10}^{-3}$$

= 3.83 * 50 * 180 * $$\:{10}^{-3}$$ = 34.507 kN.

$\:{v}_{\:u}$ = 22.36 kN ˂˂ $\:{v}_{\:max}$ = 34.507 kN case A (ok).

$\:{v}_{\:u}$ = 30.51 kN ˂˂ $\:{v}_{\:max}$ = 34.507 kN case B (ok).

### Shear design according to ACI 318 − 19

b = 50 mm, t = 200 mm, d = 180 mm, $\:{f}_{\boldsymbol{y}\:}$**=** 460 MPa, $\:{f}_{\boldsymbol{c}\:}\boldsymbol{{\prime\:}}$**=** 30 MPa, $\:\varnothing\:$ = 0.75.

#### Solution

1. Concrete contribution

$$\:{v}_{c}=\varnothing\:*\:\sqrt{{f}_{\boldsymbol{c}\:}\boldsymbol{{\prime\:}}}*b*d$$ = 0.75*5.44*50*180 $$\:*{10}^{-3}$$= 36.7 kN.

2. Shear reinforcement contribution.

$$\:{v}_{s}=\frac{{A}_{v}*\:{f}_{y}*d*Sin\:\theta\:}{s}$$ = 0.335*460*180$$\:*{10}^{-3}$$ = 27.74 kN.

3. Nominal shear capacity

$$\:{v}_{n}={v}_{c}+\:{v}_{s}$$ = 36.7 + 27.74 = 64.38 kN.

4. Minimum shear reinforcement

$$\:\left(\frac{{A}_{v}}{s}>\frac{50\:b}{{f}_{y}}\right)$$.

$$\:\frac{{A}_{v}}{s}$$ = $$\:\frac{100.5}{150}$$ = 0.67 mm^2^/mm.

$$\:\frac{50\:b}{{f}_{y}}$$ = $$\:\frac{50*50}{460}$$ = 5.435 mm^2^/mm.

For Ø 8 single leg A = 50.3 mm^2^.

$$\:\frac{{A}_{v}}{s}$$ = $$\:\frac{50.3}{150}$$ = 0.335 mm^2^/mm.

S= $$\:\raisebox{1ex}{$A$}\!\left/\:\!\raisebox{-1ex}{$\raisebox{1ex}{${A}_{v}$}\!\left/\:\!\raisebox{-1ex}{$s$}\right.$}\right.$$= 50.27/5.435 = 9.3 mm.

5. Maximum shear strength

($$\:{v}_{n}\le\:0.66\sqrt{{f}_{\boldsymbol{c}\:}\boldsymbol{{\prime\:}}}*b*d)$$

$$\:{v}_{u\:max}=0.66*\sqrt{30}*50*180$$ = 32.3kN.

$$\:{v}_{n}$$ = 64.38 kN ˃ $$\:{v}_{u\:max}$$= 32.3 kN.

### Shear design according to EN 92

*Given data for Rectangular beam section*.

b = 50 mm, t = 200 mm, d = 180 mm, $\:{f}_{\boldsymbol{c}\:}\boldsymbol{{\prime\:}}$**=** 30 MPa, $\:{f}_{\boldsymbol{y}\boldsymbol{k}\:}$**=** 500 MPa, $\:\mathrm{cot}\theta\:$ = 2.5, $\:{\rho\:}_{\lambda\:}$=0.015.

#### Solution

1. Concrete contribution

$$\:{V}_{c}=\frac{0.0525}{{\gamma\:}_{c}}*{\left({f}_{c}^{{\prime\:}}\right)}^{\raisebox{1ex}{$2$}\!\left/\:\!\raisebox{-1ex}{$3$}\right.}*\left(1.6-d\right)\left(1.2+40{\rho\:}_{\lambda\:}\right)*b*d$$

$$\:{V}_{c}=\frac{0.0525}{1.5}*{\left(30\right)}^{\raisebox{1ex}{$2$}\!\left/\:\!\raisebox{-1ex}{$3$}\right.}*\left(1.6-0.18\right)\left(1.2+40*0.015\right)*0.05*0.18$$ = 7.77 kN.

2. Shear reinforcement contribution

$$\:{V}_{s}={0.9*\rho\:}_{v}*{f}_{vy}*b*d$$

$$\:{V}_{s}=0.9*8.76*{10}^{-4}*500*50*180$$ = 3.55 kN.

3. Total shear strength

$$\:{v}_{n}={v}_{c}+\:{v}_{s}$$ = 7.77 + 3.55 = 11.32 kN.

4. Minimum shear reinforcement

$$\:\frac{{A}_{sw}}{s}\ge\:{\rho\:}_{w\:min}*b$$

$$\:{\rho\:}_{w\:min}=0.08*\frac{\sqrt{{f}_{ck}}}{{f}_{yk}}$$ = 0.08*$$\:\frac{\sqrt{30}}{500}$$ = 8.76*10^− 4^.

$$\:{\rho\:}_{w\:min}*b$$ = 0.000876 * 50 = 0.0438 mm^2^/mm.

$$\:\frac{{A}_{sw}}{s}$$ = $$\:\frac{50.3}{150}$$ = 0.335 mm^2^/mm.

$\:\frac{{A}_{sw}}{s}\ge\:{\rho\:}_{w\:min}*b=\:$ 0.263 mm^2^/mm ok.

5. Maximum shear reinforcement

$$\:\mathrm{cot}\theta\:=2.5\:,\:v=0.6\:(1-\frac{{f}_{ck}}{250}\:)\:=0.528$$

α_c_*​b_w_*​z*v*f_cd_​=1.0 × 50 × 162 × 0.528 × 20 = 85,536 N.

$$\:\mathrm{cot}\theta\:+\mathrm{tan}\theta\:$$ =2.5 + 0.4 = 2.9.

$$\:{v}_{u\:max}=\frac{85536}{2.9}$$ = 29.5 kN.

$\:{v}_{n}=$11.32 kN ˂˂ $\:{v}_{u\:max}=\:$29.5 kN ok.

### Shear design according to CSA

*Given data for Rectangular beam section*.

b_w_ = 50 mm, t = 200 mm, d = 180 mm, $\:{f}_{\boldsymbol{c}\:}\boldsymbol{{\prime\:}}$**=** 30 MPa, $\:{\uplambda\:}=1$, Ø_c_ = 0.18, ꞵ = 1+$\:\sqrt{\frac{200}{d}}$ = 1+$\:\sqrt{\frac{200}{180}}$ = 2.054, $\:{f}_{yv}=400MPa$, ν = 0.6 (1- $\:\frac{{f}_{ck}}{250}$)= 0.528, z = 0.9 d=162 m, assume $\:\varnothing\:$ 8.

#### Solution

1. Concrete contribution

$$\:{v}_{c}={\varnothing\:}_{c}*\beta\:*\lambda\:*\:\sqrt{{f}_{\boldsymbol{c}\:}\boldsymbol{{\prime\:}}}*{b}_{w}*d$$

$$\:{v}_{c}=0.18*2.054*1*\:\sqrt{30}*9000$$ = 18.23 kN.

2. Shear reinforcement contribution

$$\:{v}_{s}={A}_{v}\mathrm{*}{f}_{yv}\mathrm{*}\frac{d}{s}\:\:=\:50.3\mathrm{*}400\mathrm{*}\frac{180}{150}\:=\:24.13\:\mathrm{k}\mathrm{N}$$

3. Total shear strength

$$\:{v}_{r}={v}_{c}+\:{v}_{s}$$ = 18.23 + 24.13 = 42.36 kN.

4. Minimum shear reinforcement

$$\:\frac{{A}_{v}}{s}=\frac{50.3}{150}=0.335\ge\:\frac{0.06\sqrt{{f}_{\boldsymbol{c}\:}\boldsymbol{{\prime\:}}}}{\:{f}_{y}}*{b}_{w}=\:0.041{\mathrm{m}\mathrm{m}}^{2}/\mathrm{m}\mathrm{m}\:$$ ok.

5. Maximum shear strength

$$\:{v}_{max}={\propto\:}_{c}.z\:\mathrm{v}\:{b}_{w}{f}_{\boldsymbol{c}\:}\boldsymbol{{\prime\:}}.\:\frac{\mathrm{cot}\theta\:}{1+{\mathrm{cos}}^{2}\theta\:}$$

$$\:{v}_{max}=128.3*1.341$$ = 172 kN.

$\:{v}_{r}=$ 42.36 kN ˂ $\:{v}_{max}=$ 172 kN ok.

### Shear design according to BS 8110

*Given data for Rectangular beam section*.

b_v_ = 50 mm, t = 200 mm, d = 180 mm, $\:{f}_{\boldsymbol{c}\boldsymbol{u}\:}$ = 30 MPa,

$$\:{\gamma\:}_{m}=1.25,\:\mathrm{A}\mathrm{s}=\:150\:\mathrm{m}\mathrm{m}$$^2^.

#### Solution

1. Concrete contribution

$$\:{v}_{c}=\frac{0.79}{{\gamma\:}_{m}}\:.\:{\left(\frac{100\:{A}_{s}}{{b}_{v}d}\right)}^{\raisebox{1ex}{$1$}\!\left/\:\!\raisebox{-1ex}{$3$}\right.}.{\left(\frac{400\:}{d}\right)}^{\raisebox{1ex}{$1$}\!\left/\:\!\raisebox{-1ex}{$4$}\right.}.{\left(\frac{\:{f}_{cu}}{25}\right)}^{\raisebox{1ex}{$1$}\!\left/\:\!\raisebox{-1ex}{$3$}\right.}.\mathrm{b}.\mathrm{d}$$

$$v_{c} = \frac{{0.79}}{{1.25}} \cdot \left( {\frac{{100*150}}{{50*180}}} \right)^{{{\raise0.7ex\hbox{$1$} \!\mathord{\left/ {\vphantom {1 3}}\right.\kern-\nulldelimiterspace} \!\lower0.7ex\hbox{$3$}}}} \cdot \left( {\frac{{400~}}{{180}}} \right)^{{{\raise0.7ex\hbox{$1$} \!\mathord{\left/ {\vphantom {1 4}}\right.\kern-\nulldelimiterspace} \!\lower0.7ex\hbox{$4$}}}} \cdot \left( {\frac{{~30}}{{25}}} \right)^{{{\raise0.7ex\hbox{$1$} \!\mathord{\left/ {\vphantom {1 3}}\right.\kern-\nulldelimiterspace} \!\lower0.7ex\hbox{$3$}}}} \cdot 50.180{\text{ }} = {\text{ }}8.69{\text{ }}kN$$

2. Shear reinforcement contribution

$$\:{v}_{s}=0.87{\rho\:}_{v}.\:{f}_{vy}.\mathrm{b}.\mathrm{d}$$

$$\:{\rho\:}_{v}\:=\:\frac{\:{A}_{sv}}{{b}_{v}s}$$

For 2 leg Ø 8 $\:{A}_{sv}$ =2* $\:\frac{\pi\:*{8}^{2}}{4}$ = 100.53 mm^2^

$$\:{\rho\:}_{v}\:=\:\frac{100.53}{50*150}\:=\:0.0134$$

$$\:{v}_{s}=0.87{\rho\:}_{v}.\:{f}_{vy}.\mathrm{b}.\mathrm{d}$$

= 0.87* 0.0134*250*50*180 = 26.24 kN.

3. Total shear strength

$$\:{v}_{r}={v}_{c}+\:{v}_{s}$$ = 8.69 + 26.24 = 34.93 kN.

4. Minimum shear reinforcement

$$\:\frac{\:{A}_{sv}}{{b}_{v}s}=0.0134\:\ge\:\:\frac{\:0.4}{{f}_{yv}}=\frac{\:0.4}{250}=0.00169$$ok.

5. Maximum shear strength

$$\:{v}_{max}={\tau\:}_{c\:max}$$.b.d.

$\:{\tau\:}_{c\:max}$= 5 N/mm^2^ (from Tables 3, 4, 5, 6, 7 and 8).

$$\:{v}_{max}=5$$*9000 = 45 kN.

$\:{v}_{r}=$34.93 kN ˂ $\:{v}_{max}=\:$45 kN ok.

### Shear design according to JSCE

*Given data for Rectangular beam section*.

b_w_ = 50 mm, t = 200 mm, d = 180 mm, *f*_c_′ = 30 MPa,

1. Concrete contribution

$$v_{c} = \alpha * \beta * \surd f_{c}^{'} * b_{w} * d$$ 

$$\:\alpha\:=1-0.2*\left(\frac{\raisebox{1ex}{$l$}\!\left/\:\!\raisebox{-1ex}{$d$}\right.-2}{4}\right)\:\:\:\:for\:\:2<\raisebox{1ex}{$l$}\!\left/\:\!\raisebox{-1ex}{$d$}\right.\le\:6$$

$$\:\raisebox{1ex}{$l$}\!\left/\:\!\raisebox{-1ex}{$d$}\right.$$ =$$\:\raisebox{1ex}{$1000$}\!\left/\:\!\raisebox{-1ex}{$180$}\right.$$ = 5.56 $$\:\le\:6$$ ok.

$$\:\alpha\:\:$$= 1 − 0.2(0.89) = 0.822

$$\:\rho\:=\frac{{A}_{s}}{{b}_{w}*d}\:=\:\frac{150}{50*180}\:=\:0.0167$$

$$\:\beta\:=1+2.5\rho\:$$ = 1 + 2.5(0.0167) = 1.0417.

$$\: v_{c} = \alpha *\beta *\surd f^{\prime}_{c} *b_{w} *d$$

= 0.822*1.0417*5.477*50*180 = 0.422kN.

2. Shear reinforcement contribution

$$\:{v}_{s}=\frac{{A}_{sv}*\:{f}_{y}*d}{s}\:=\:\frac{50.3*\:360*180}{100}\:=\:32.6\:\mathrm{k}\mathrm{N}$$

3. Total shear strength

$$\:{v}_{r}={v}_{c}+\:{v}_{s}$$ = 0.422 + 32.6 = 33.02 kN.

4. Minimum shear reinforcement

$$\:\frac{\:{A}_{sv}}{{b}_{v}s}=0.01006\:\ge\:\:\frac{\:0.062\surd\:\mathrm{f}\mathrm{c}\:{\prime\:}\text{}}{{f}_{y}}=\frac{\:0.062*5.477}{360}=0.000943$$ok.

5. Maximum shear strength

$$\:{v}_{max}=$$ 0.2*$$\:{b}_{w}*d*fc{\prime\:}$$= 0.2*50*180*30*10^− 3^=45kN.

Table 4 provides a comprehensive overview of shear capacity predictions for reinforced concrete (RC) beams according to various international design codes, including ECP 203–2020, the American Concrete Institute, Eurocode 2, the Canadian Standards Association, the British Standards Institution, and the Japan Society of Civil Engineers. Two types of beams are examined: non-slender beams and slender beams, differentiated by their geometric proportions. For each case, key parameters such as beam width (b), effective depth (d), and concrete compressive strength (f_c_′) are defined, followed by the calculated contributions of concrete (V_c_ ​) and shear reinforcement (V_s_ ​), leading to the total shear capacity (V_u_). The table also includes the minimum shear reinforcement ratio A_v_/(b_w_s), the maximum allowable shear capacity (V_max_), and the utilization ratio (V_u_/V_max_), which indicates the level of safety or conservatism in each code. For non-slender beams, major variations are seen among the codes, with the American Concrete Institute and Japan Society of Civil Engineers forecasting significantly greater shear capabilities than Eurocode 2 and British Standards Institution. This is evidenced by a mean usage ratio of 0.68 and a high coefficient of variation (44%), signifying substantial dispersion and variability among design methodologies. Conversely, for slender beams, the anticipated values are typically lower and more uniform, with a higher mean utilization ratio of 0.78, accompanied by a greater coefficient of variation (81%), indicating heightened sensitivity of thin beam behavior to the selected design model. The table underscores the impact of code formulation on shear design predictions, specifically on the consideration of size effect, reinforcement contribution, and maximum shear limits by each code. The observed variations highlight the necessity for meticulous selection and comparison of design provisions for assessing shear behavior in reinforced concrete beams, particularly in research endeavors focused on standardization or code calibration.

**Fig. 3:**
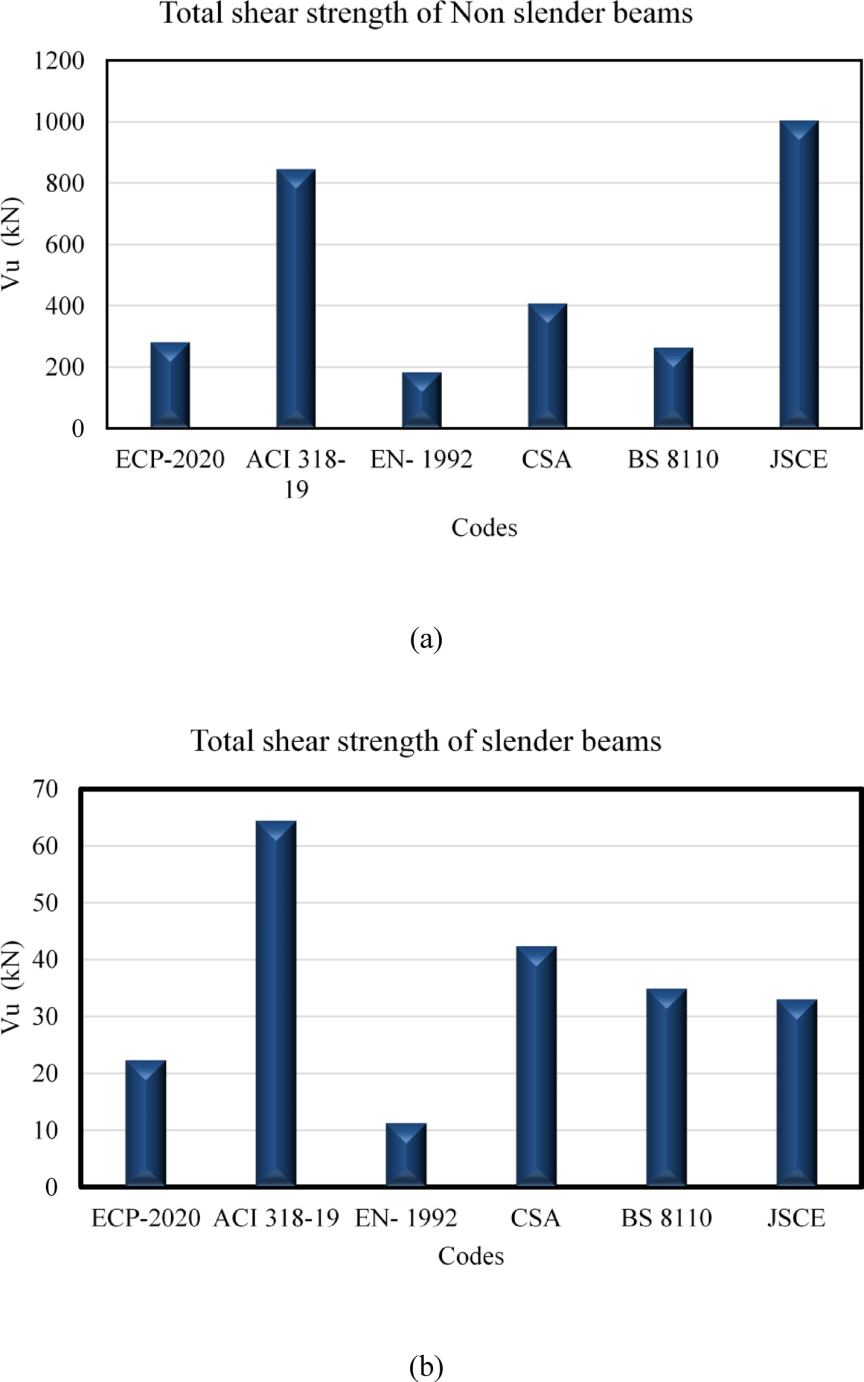
Total shear strength of Non slender versus slender beams according to different codes.

Figures (3a and b) illustrate a comparative assessment of the total shear strength (V_u_) forecasted by various international design codes for both non-slender and slender reinforced concrete (RC) beams, encompassing ECP 203–2020, the American Concrete Institute, Eurocode 2, the Canadian Standards Association, the British Standards Institution, and the Japan Society of Civil Engineers. A significant difference is noted in the estimated shear capacity for non-slender beams. The Japan Society of Civil Engineers and American Concrete Institute produce the highest values of (V_u_), suggesting less conservative estimates, while Eurocode 2 offers the lowest estimate, indicating a more conservative methodology. The Canadian Standards Association and British Standards Institution occupy an intermediate range, whereas ECP 203–2020 presents mild forecasts compared to BS 8110. This variety underscores the substantial impact of diverse modeling approaches, especially on how each code addresses shear transfer mechanisms, including aggregate interlock, dowel action, and size effect.

Conversely, for narrow beams, while the total magnitude of (V_u_) is markedly diminished due to decreased section dimensions and increased slenderness, the relative trends among the codes remain consistent. The American Concrete Institute consistently forecasts the highest shear capacity, succeeded by the Canadian Standards Association, whilst Eurocode 2 consistently produces the lowest results. Nonetheless, the disparities among codes diminish in comparison to the non-slim scenario, indicating a comparatively more uniform predictive framework for slender members.

The results indicate that code-based predictions of shear strength are significantly influenced by the chosen formulation, exhibiting considerable diversity among design standards. This underscores the necessity for meticulous interpretation when juxtaposing data or choosing a design code, especially in research settings focused on evaluating the dependability, conservatism, and possible harmonization of shear design rules.

**Fig. 4:**
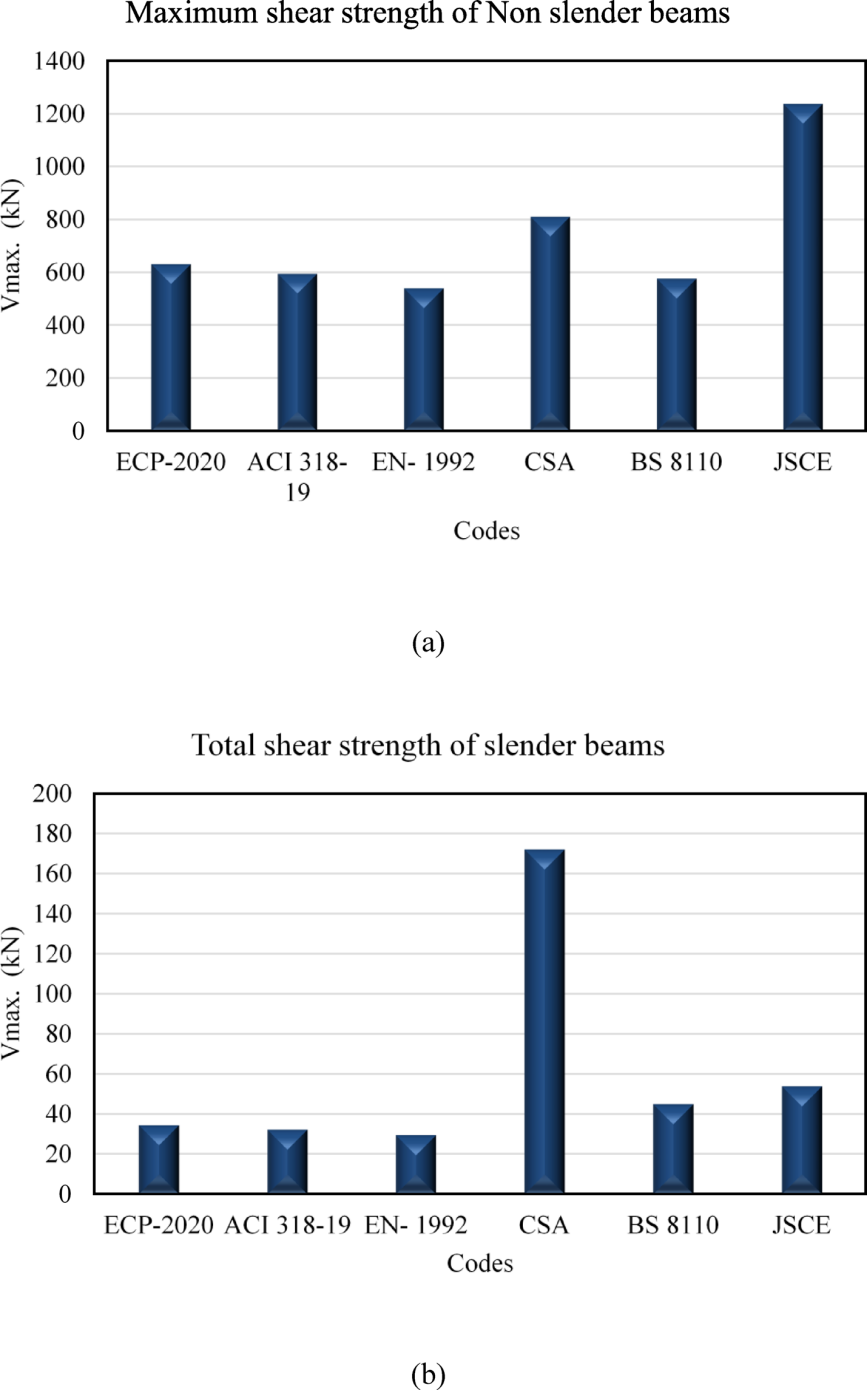
Maximum shear strength of Non slender versus slender beams according to different codes.

**Fig. 5:**
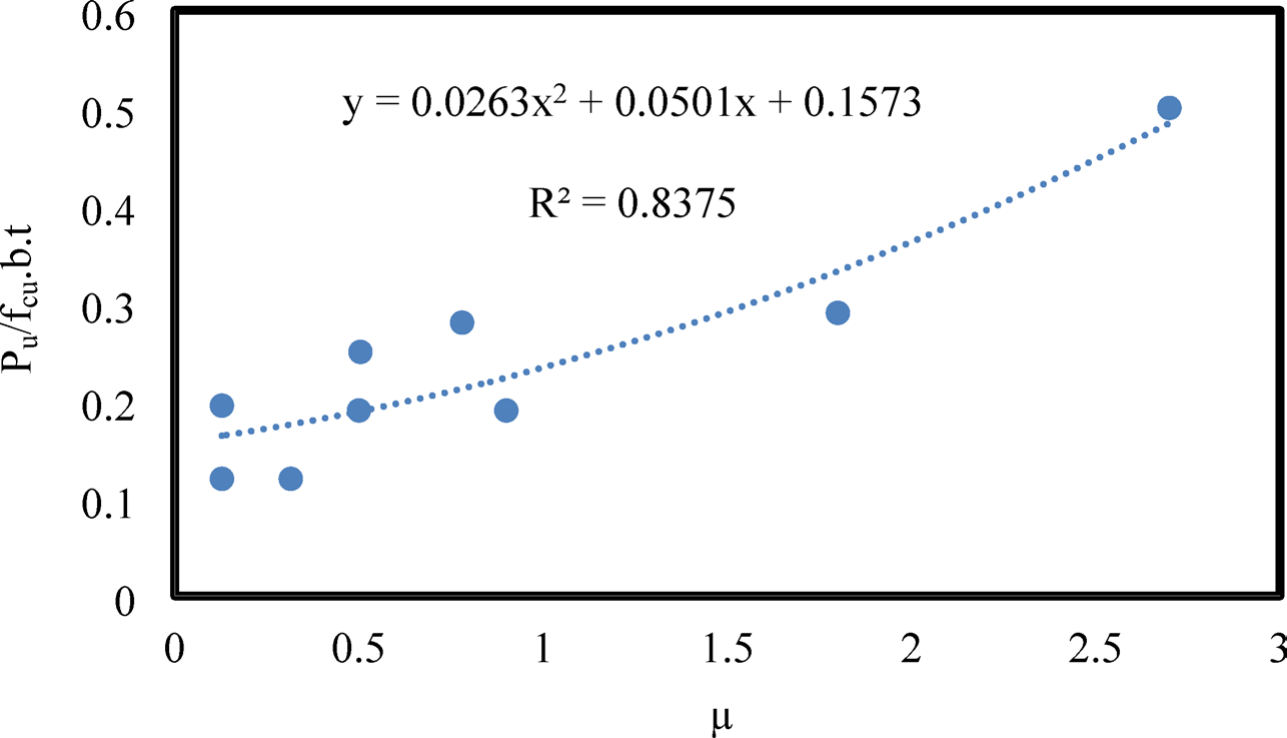
Distribution of different µ versus ultimate load Pu of presented beams.

Fig. (4a and b) depicte the fluctuations in the maximum shear capacity (V _max_) as forecasted by various international design codes for non-slender reinforced concrete (RC) beams, encompassing ECP 203–2020, the American Concrete Institute, Eurocode 2, the Canadian Standards Association, the British Standards Institution, and the Japan Society of Civil Engineers. The results indicate substantial variances among the codes in assessing the upper limit of shear resistance dictated by concrete crushing or diagonal compression failure. The Japan Society of Civil Engineers offers the most optimistic prediction of (V_max_), reflecting a less conservative methodology in establishing the concrete compression limit, succeeded by the Canadian Standards Association. Conversely, Eurocode 2 and the American Concrete Institute produce relatively lower numbers, indicating more conservative assumptions about the maximum permissible shear stress in concrete. The British Standards Institution and ECP 203–2020 occupy an intermediate range, indicating a compromise between safety and cost-effectiveness. The deviations are mainly due to discrepancies in the modeling of the shear compression mechanism, encompassing the handling of strut inclination, concrete softening, and strength reduction factors. Certain standards, like Eurocode 2, expressly integrate safety issues and stress limitations, whilst others, such as those from the Japan Society of Civil Engineers, utilize formulations that may permit greater exploitation of concrete compressive strength. As illustrated in Fig. 5, the estimation of maximum shear capacity is significantly reliant on code specifications, with important consequences for structural safety and design prudence. This underscores the significance of comprehending the foundational assumptions of any design standard when conducting comparison analyses or choosing a suitable code for practical applications.

**Table 4:** A comprehensive overview of shear capacity predictions for reinforced concrete (RC) slender and non-slender beams according to various international design codes.

Parameter	ECP-2020	ACI 318 − 19	EN −1992	CSA	BS 8110	JSCE	Non slender beams	Slender beams
b (mm)	300							-
d (mm)	550							-
F_c_’ (MPa)	30							-
V_c_(kN)	144.5	678	110	260.8	117	798		-
V_s_(kN)	138.23	169	74.3	147.4	148	207		-
V_u_(kN)	282.8	847	184.3	408.2	265	1005		-
A_v min_.(mm^2^/mm)	1.047	0.67	0.67	0.246	0.0022	0.67		-
V_max_(kN)	632.62	595	541	811.1	577.7	1237.5		-
V_u_/V_max_	0.45	1.42	0.34	0.50	0.46	0.81		-
Mean	0.68							
Coefficient of variation (%)	0.44							
b (mm)	50						-	✓
d (mm)	180						-	✓
F_c_’ (MPa)	30						-	✓
V_c_(kN)	7.887	36.7	7.77	18.23	8.69	0.422	-	✓
V_s_(kN)	14.476	27.74	3.55	24.13	26.24	32.6	-	✓
V_u_(kN)	22.36	64.38	11.32	42.36	34.93	33	-	✓
A_v min_.(mm^2^/mm)	0.335	0.335	0.335	0.335	0.0134	0.01006	-	✓
V_max_(kN)	34.507	32.3	29.5	172	45	54	-	✓
V_u_/V_max_	0.64	2	0.38	0.25	0.78	0.61	-	✓
Mean	0.78						-	✓
Coefficient of variation (%)	0.81						-	✓

## Analytical predicting ultimate load with different reinforcement ratios

### Deriving a new equation to calculate the ultimate load

In this study the relationship between ultimate load (P_u_) at different shear reinforcement ratios (µ) is illustrated in Fig. 5 For every constant, a polynomial trend-line is fitted, providing a high R-squared value. A data point located at the beginning of the polynomial trend line indicates that stage I demonstrated a slowing escalation until the shear reinforcement ratio achieved 1.8%. Stage II demonstrated a sharp increase with a shear reinforcement ratio of 2.7%.

A data point that is above the trend line indicates a conservative prediction, whereas a data point that is below the trend line indicates an un-conservative one. It is evident that the closer the R^2^ number is to 1, the more precise and palatable the results. The result of R^2^ in the current study is 0.83 which can be accepted. The currently available equation to directly predict the ultimate load of tested beams with different shear reinforcement ratios is mostly an empirical equation. The ultimate load of slender beams (P_u_) with different reinforcement ratios (µ) can be calculated from the proposed Eq. 47 and Eq. 48.

47 $$y{\text{ }} = {\text{ } y{\text{ }} = {\text{ }}0.0263x^{2} + {\text{ }}0.0501x{\text{ }} + {\text{ }}0.1573 }0.0263x^{2} + {\text{ }}0.0501x{\text{ }} + {\text{ }}0.1573$$

48 $$Pu = {\text{ }}fcu.b.t{\text{ }}\left( {0.0263{\text{ }}\mu ^{2} + {\text{ }}0.0501{\text{ }}\mu {\text{ }} + {\text{ }}0.1573} \right)$$

Where y refers to the ultimate load (P_u_), the variable x in Eq. (47) refers to the shear reinforcement ratios (µ) of the tested beams in this study, b is the tested beam width, t is the beam thickness and f_cu_ is the concrete compressive strength. By considering all the beams included in the database with different reinforcement ratios, the proposed procedure gave a good correlation with the experimental results. By considering all the beams included in the database with different reinforcement ratios, the proposed procedure gave a good correlation with the experimental results.

### Application of the proposed equation on the previous studies

**Fig. 6:**
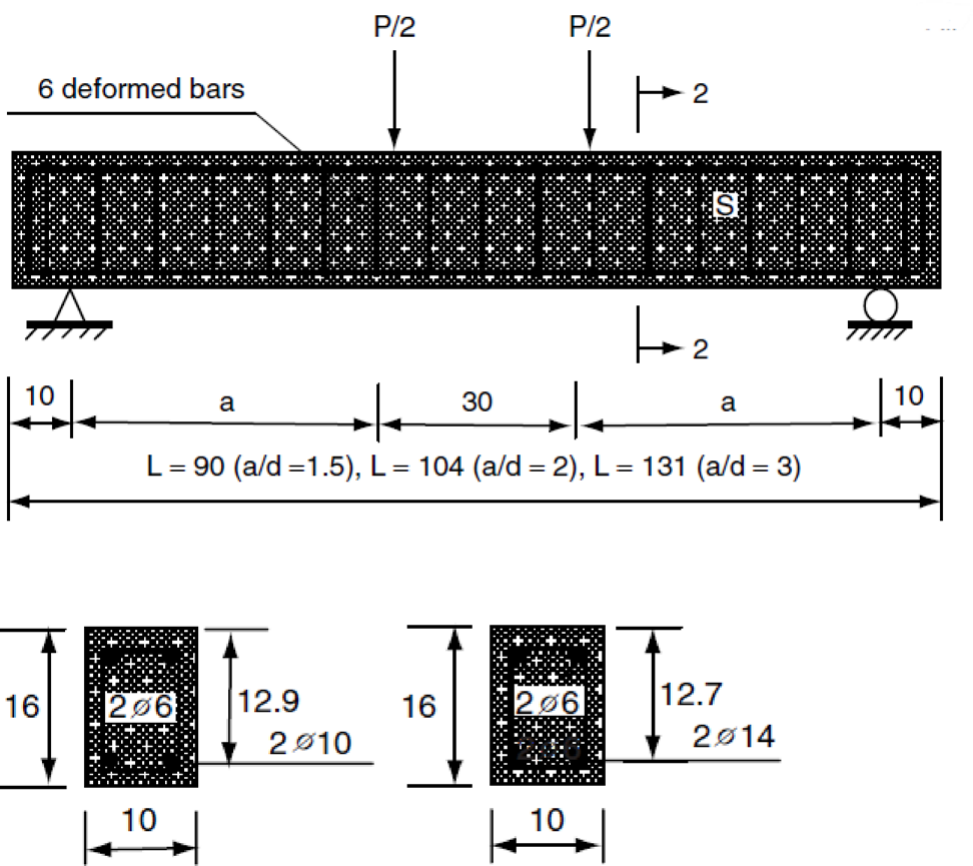
Dimensions and reinforcement of the test beams of group W (44 MPa, 65 MPa and 86 MPa)^26^.

**Fig. 7:**
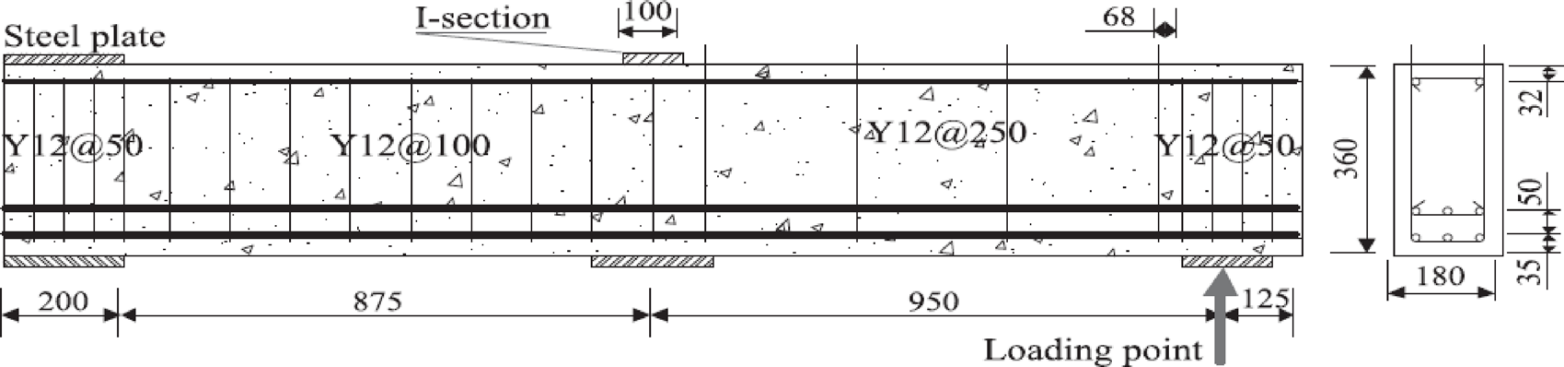
Details of RC specimen D 3.1^38^.

**Fig. 8:**
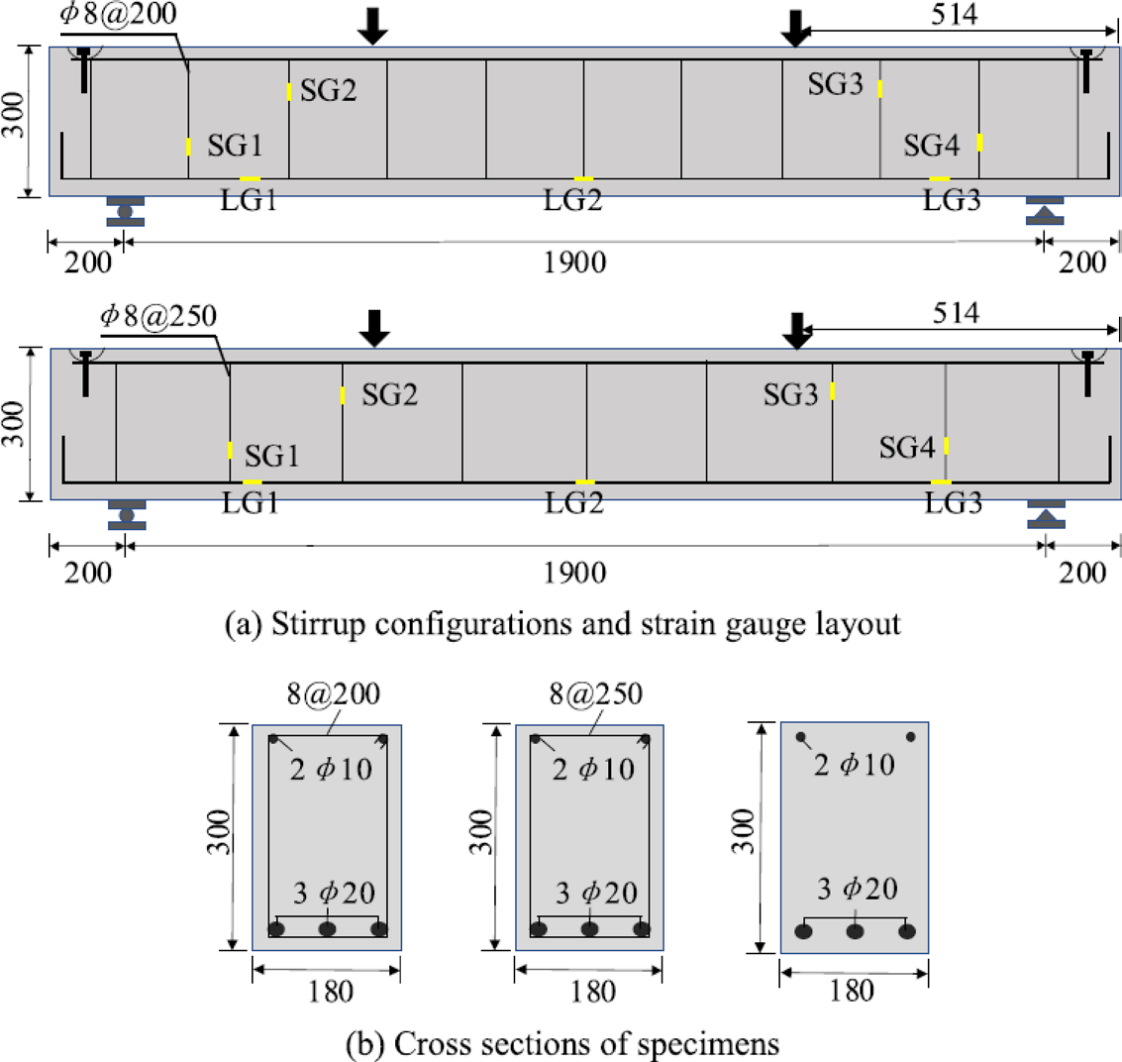
Geometry and reinforcement details of the beams (unit: mm)^39^.

The proposed Eq. (48) has been applied as predicted by^26,39,39^ in order to verify the accuracy of the test results with the proposed values as illustrated in Table 5. The effects of transverse reinforcement on the crack patterns, the ultimate carrying capacity and the ductility of beams made of high strength concrete and normal strength concrete for comparison purposes have been investigated by^26^. Twenty-six reinforced concrete beams with and without transverse reinforcement were tested using different shear-span to depth ratios (a/d = 1.5, 2.0 and 3.0) and different compressive strengths of concrete (44 MPa, and 86 MPa). The transverse reinforcement for the beams in group W (44 W, and 86 W) was the same and consisted of stirrups with a 6 mm diameter placed 90 mm apart. The reinforcement ratio of the reinforced concrete beams of^26^ has been calculated by using Eq. 1 in the current study. The dimensions and reinforcement of the tested beams for^26^ were shown in Fig. 6. Three groups of specimens were created. Three beams of 1410, 1780, and 2150 mm in length were used in each group; these lengths corresponded to a/d values of 1.9, 2.5, and 3.1, respectively as shown in^38^. Details of the specimen was as illustrated in Fig. 7. The main test variables were shear reinforcement type (deformed or plain round bar, characterized by strong or weak bond, respectively) and a/d. Each specimen was identified by a letter (N, D or R) and a number (1.9, 2.5 or 3.1). The letter denotes the type of transverse reinforcement, N for beams without stirrup, D for deformed bar, and R for plain round bar. Through tests, numerical analyses, and theoretical research, the impact of steel reinforcement set up with varying strengths following various high temperatures on the test beams’ shear performance a total of 20 reinforced concrete (RC) beams was examined in order to identify the main variables influencing the beams’ shear performance. High-strength and normal-strength reinforced concrete beams with a stirrup spaced 200 mm apart are indicated by the NS-200 and HS-200 series beams as shown in^39^. The geometry and reinforcement details of the beams were shown in Fig. 8. By considering all the beams included in the database, the proposed procedure gave a good correlation with the experimental results.

**Table 5:** Proposed bending capacity versus experimental results for^26,39,39^ and current study.

Ref.	Specimen	b (mm)	t (mm)	f_cu_ (*N*/mm^2^)	µ	a (mm)	$\:\frac{a}{d}$	$\:{\mathrm{P}}_{\mathrm{u}\:\mathrm{e}\mathrm{x}\mathrm{p}\:}\:\left(\mathrm{N}\right)$	*P*_u prop_ (*N*)	$\:\frac{{\mathrm{P}}_{\mathrm{u}\:\mathrm{e}\mathrm{x}\mathrm{p}}}{{\mathrm{P}}_{\mathrm{u}\:\mathrm{p}\mathrm{r}\mathrm{o}\mathrm{p}}}$
^26^	A44-1.5w	100	160	44	0.63	200	1.5	148.2*10^3^	140.3*10^3^	1.05
	B44-1.5w	100	160	44	0.63	200	1.5	173.7*10^3^	140.3*10^3^	1.2
	A44-2w	100	160	44	0.63	270	2	104.5*10^3^	140.3*10^3^	0.7
	B44-2w	100	160	44	0.63	270	2	140.9*10^3^	140.3*10^3^	1
	B44-3w	100	160	44	0.63	405	3	100.9*10^3^	140.9*10^3^	0.7
	B86-1.5w	100	160	86	0.63	200	1.5	192.2*10^3^	274*10^3^	0.7
^38^	D 3.1	180	360	40.3	0.42	930	3.1	492.4*10^3^	478*10^3^	1
^39^	Ns-200-a	180	3000	51.3	0.28	414	2	489 *10^3^	531.7*10^3^	0.9
	Hs-200-a	180	300	51.3	0.28	414	2	489.2*10^3^	531.7*10^3^	0.9
	Hs-250-a	180	300	51.3	0.22	414	2	378.9*10^3^	471*10^3^	0.8
	Ns-200-b	180	300	50.62	0.28	414	2	469*10^3^	473.9*10^3^	1
	Hs-200-b	180	300	50.62	0.28	414	2	539.76*10^3^	473.9*10^3^	1.1
	Hs-250-b	180	300	50.62	0.22	414	2	469*10^3^	464*10^3^	1.09

### Calculations of the proposed equation on the previous studies

1. Specimen A44-1.5 W

P_u exp_. =148.2 * 10^3^ N a = 200 mm

P_u prop. =_ $$\:44*100*160[0.0263{\left(0.63\right)}^{2}+0.0501\left(0.63\right)+0.1573=140.3*{10}^{3}$$N

2. Specimen B44-1.5 W

P_u exp_. =173.7 * 10^3^ N a = 200 mm

P_u prop. =_ $$\:44*100*160[0.0263{\left(0.63\right)}^{2}+0.0501\left(0.63\right)+0.1573=140.3*{10}^{3}$$N

3. Specimen A44-2 W

P_u exp_. =104.5 * 10^3^ N a = 270 mm

P_u prop. =_ $$\:44*100*160[0.0263{\left(0.63\right)}^{2}+0.0501\left(0.63\right)+0.1573=140.3*{10}^{3}$$N

4. Specimen B44-2 W

P_u exp_. =140.9 * 10^3^ N a = 270 mm

P_u prop. =_ $$\:44*100*160[0.0263{\left(0.63\right)}^{2}+0.0501\left(0.63\right)+0.1573=140.3*{10}^{3}$$N

5. Specimen B44-3 W

P_u exp_. =100.9 * 10^3^ N a = 405 mm

P_u prop. =_ $$\:44*100*160[0.0263{\left(0.63\right)}^{2}+0.0501\left(0.63\right)+0.1573=140.3*{10}^{3}$$N

6. Specimen B86-1.5 W

P_u_=192.2 * 10^3^ N a = 200 mm

P_u prop. =_ $$\:86*100*160[0.0263{\left(0.63\right)}^{2}+0.0501\left(0.63\right)+0.1573=274*{10}^{3}$$N

7. Specimen D 3.1

P_u exp_. =492.4 * 10^3^ N a = 930 mm

P_u prop. =_ $$\:40.3*180*360[0.0263{\left(0.42\right)}^{2}+0.0501\left(0.42\right)+0.1573=478*{10}^{3}$$N

8. Specimen Ns-200-a

P_u_=489.01 * 10^3^ N a = 414 mm

P_u prop. =_ $$\:51.3*180*300[0.0263{\left(0.28\right)}^{2}+0.0501\left(0.28\right)+0.1573=531.7*{10}^{3}$$N

9. Specimen Hs-200-a

P_u_=489.24 * 10^3^ N a = 414 mm

P_u prop. =_ $$\:51.3*180*300[0.0263{\left(0.28\right)}^{2}+0.0501\left(0.28\right)+0.1573=531.7*{10}^{3}$$N

10. Specimen Hs-250-a

P_u_=378.9 * 10^3^ N a = 414 mm

P_u prop. =_ $$\:51.3*180*300[0.0263{\left(0.22\right)}^{2}+0.0501\left(0.22\right)+0.1573=471*{10}^{3}$$N

11. Specimen Ns-200-b

P_u exp_=469.14 * 10^3^ N a = 414 mm

P_u prop. =_ $$\:50.62*180*300[0.0263{\left(0.28\right)}^{2}+0.0501\left(0.28\right)+0.1573=473.9*{10}^{3}$$N

12. Specimen Hs-200-b

P_u exp_=539.76 * 10^3^ N a = 414 mm

P_u prop. =_ $$\:50.62*180*300[0.0263{\left(0.28\right)}^{2}+0.0501\left(0.28\right)+0.1573=473.9*{10}^{3}$$N

13. Specimen Hs-250-b

P_u exp_ =509.7 * 10^3^ N a = 414 mm

P_u prop. =_ $$\:54.6*180*300[0.0263{\left(0.22\right)}^{2}+0.0501\left(0.22\right)+0.1573=500.3*{10}^{3}$$N

## Conclusion

This study comprehensively examines the shear performance and design specifications of slender reinforced concrete beams as delineated by various international standards, including ECP, ACI, Eurocode, CSA, BS, and JSCE. The analysis indicates considerable differences in the methods utilized for assessing shear capacity, minimum reinforcement requirements, and serviceability criteria across these codes. These differences can lead to reinforcing demands that are too conservative or not adequate, affecting the structural integrity and safety of slender beams. The principal findings underscore the necessity of adhering to relevant design guidelines that incorporate contemporary research and practical considerations. By integrating these criteria into design procedures, engineers can enhance the resilience and efficiency of slender beams under diverse loading conditions. The study emphasizes the necessity for standardized protocols to unify shear design methodologies, as discrepancies in code regulations can hinder endeavors to achieve efficiency and safety in structural design. This research proves many conclusions as below:

1. The study emphasizes the necessity of complying with pertinent codes, as they embody the most recent research and practical issues in the discipline. Integrating these standards into design procedures enables engineers to improve the structural integrity and durability of RC slender beams under diverse loading circumstances.
2. To keep improving the safety and efficiency of slender concrete structures in contemporary construction, future research should focus on establishing efficient design methods that incorporate advancements in materials and technology.
3. ACI 318 and other codes mandate a minimum tension reinforcement ratio of 0.2% of the gross cross-sectional area, whereas BS 8110 and CSA standards provide almost the same minimum reinforcement ratios, with slightly different exact values, to guarantee control of cracks and ductility.
4. Crack width restrictions are affected by exposure circumstances; ACI and EN standards prescribe maximum crack widths of 0.1% or 0.2% of the cross-sectional dimensions in order to provide durability against disturbance to the environment.
5. Influence of Beam Slenderness: Slender beams, characterized by shear span-to-depth ratios exceeding typical limits (e.g., a/d > 2.5 per ACI, a/h > 3 per Eurocode), exhibit reduced shear strengths across all codes with increased variability (up to 81% coefficient of variation). This highlights the critical need for careful design consideration of slenderness effects in shear evaluation.
6. The evaluated codes show significant differences in predicted shear strengths. For instance, ACI 318-19 generally predicts the highest shear capacities (up to V_u_/V_max_ = 1.42 for non-slender beams), reflecting a less conservative approach, while Eurocode 2 provides the lowest and most conservative values (V_u_/V_max_ as low as 0.34). Other codes fall between these two extremes.
7. Differences exist among codes on minimum tension and shear reinforcement ratios, influencing crack widths and ductility. Codes like ACI 318 prescribe minimum tension reinforcement of 0.2% of the gross section, while BS 8110 and CSA offer slightly varying values around 0.22%. Crack width limits vary but commonly range between 0.1 mm and 0.2 mm depending on exposure, directly affecting durability.
8. The study underscores the necessity of harmonizing shear design provisions internationally, particularly for slender beams, to reduce discrepancies that may compromise safety or lead to inefficiencies. Future work should focus on experimental validation under various loading and environmental conditions, development of advanced predictive models, and integration of emerging materials and technologies.
9. Given the variability in design approaches and their impact on safety margins, it is vital for practitioners to select and apply the relevant code carefully, considering project-specific requirements and regional practices. For slender beams, a conservative or combined approach, supported by numerical or experimental validation, is recommended to address uncertainties in shear capacity predictions.

## Recommendations for future research

1. Standard Integration: Initiatives must be undertaken to align international shear design regulations to minimize inconsistencies, promote global engineering procedures, and improve safety margins across various construction contexts.
2. Additional Investigation into Material Innovations: Explore innovative, sustainable, and more efficient repairing materials that enhance shear resistance in RC slender beams, particularly under impact and high loading situations, while adhering to environmental constraints.
3. Advancement of Sophisticated Design Instruments: Integrate contemporary modeling and numerical modeling methodologies to enhance the prediction of shear behavior, facilitating more accurate and cost-effective reinforcing procedures aligned with specific code requirements.
4. Enhanced Education and Training: Promote awareness among structural engineers regarding the nuances among different standards, emphasizing the impact of code choices on design safety and cost, particularly in international projects.
5. Empirical Validation: Conduct experimental studies to validate and refine existing code provisions, especially for slender beams under complex loading conditions, which remain areas of ongoing uncertainty.
6. Integrated Design Approach: Encourage the integration of code-specific requirements with performance-based design principles to achieve safe, economical, and sustainable slender reinforced concrete structures.

## Data Availability

The datasets used and/or analysed during the current study available from the corresponding author on reasonable request.

## Nomenclature

$\:{v}_{s}$: The shear reinforcement provides the shear strength in (kN)
$\:{v}_{c}$: The concrete’s shear strength in (kN)
D: The effective depth of the beam in (mm)
$\:{f}_{cu}$: The concrete’s typical compressive strength in (MPa)
$\:{A}_{sv}$: Area of one stirrup leg х number of legs in (mm²)
S: The spacing in mm between stirrups in (mm)
$f^{\prime}_{c}$: The compressive strength of concrete in (MPa)
$\:{f}_{y}$: The steel reinforcement’s yield strength in (MPa)
$\theta$: The angle of the concrete compression
$\phi$: The strength reduction factor
$\:{A}_{v}$: The shear reinforcement’s area in (mm^2^)
$\gamma _{c}$: The concrete material’s safety factor
$\:v_{c}$: The longitudinal steel ratio
$v_{{cz}}$: The stirrups’ nominal strength
$v_{a}$: The vertical spacing of steel strands in (mm)
$v_{d}$: The total cross sectional area of WWM in (mm)
$\phi c$: The resistance factor for concrete
$\beta$: The empirical coefficient
$\lambda$: The density factor of concrete
$\:{b}_{w}$: The web beam width in (mm)
$\gamma$: The material’s safety factor
$\alpha$: The slenderness correction factor
$\:{l}_{v}:\:$: The length of the vertical leg of the stirrups in (mm)
$V_{{cd}}$: The shear resistance of the concrete
$V_{{sd}}$: The shear resistance provided by shear reinforcement

## Declarations

### Permission to publish

The writers all concur that following acceptance, the piece will be published.

### Access to data

Upon reasonable request, the respective author can supply the datasets used and/or analyzed in the current work.
